# Interictal Gamma Event Connectivity Differentiates the Seizure Network and Outcome in Patients after Temporal Lobe Epilepsy Surgery

**DOI:** 10.1523/ENEURO.0141-22.2022

**Published:** 2022-12-15

**Authors:** Mohamad Shamas, Hsiang J. Yeh, Itzhak Fried, Jerome Engel, Richard Staba

**Affiliations:** David Geffen School of Medicine, University of California, Los Angeles, Los Angeles, CA 90095

**Keywords:** epileptic network, event connectivity, intracerebral recordings, seizure onset zone

## Abstract

Studies of interictal EEG functional connectivity in the epileptic brain seek to identify abnormal interactions between brain regions involved in generating seizures, which clinically often is defined by the seizure onset zone (SOZ). However, there is evidence for abnormal connectivity outside the SOZ (NSOZ), and removal of the SOZ does not always result in seizure control, suggesting, in some cases, that the extent of abnormal connectivity indicates a larger seizure network than the SOZ. To better understand the potential differences in interictal functional connectivity in relation to the seizure network and outcome, we computed event connectivity in the theta (4–8 Hz, ThEC), low-gamma (30–55 Hz, LGEC), and high-gamma (65–95 Hz, HGEC) bands from interictal depth EEG recorded in surgical patients with medication-resistant seizures suspected to begin in the temporal lobe. Analysis finds stronger LGEC and HGEC in SOZ than NSOZ of seizure-free (SF) patients (*p* = 1.10e-9, 0.0217), but no difference in not seizure-free (NSF) patients. There were stronger LGEC and HGEC between mesial and lateral temporal SOZ of SF than NSF patients (*p* = 0.00114, 0.00205), and stronger LGEC and ThEC in NSOZ of NSF than SF patients (*p* = 0.0089, 0.0111). These results show that event connectivity is sensitive to differences in the interactions between regions in SOZ and NSOZ and SF and NSF patients. Patients with differential strengths in event connectivity could represent a well-localized seizure network, whereas an absence of differences could indicate a larger seizure network than the one localized by the SOZ and higher likelihood for seizure recurrence.

## Significance Statement

In surgical patients with different forms of temporal lobe epilepsy, interictal event connectivity is a sensitive form of EEG functional connectivity that could be associated with synchrony of neuronal activity between brain regions. Differences in the strength of event connectivity or the lack thereof could indicate the extent of brain regions that are involved in generating seizures, which could be more numerous or larger than the clinically-defined brain area where seizures begin and correspond with the likelihood for seizure control.

## Introduction

Multimodal techniques and new signal processing approaches, such as functional connectivity analysis, have advanced the concept of epilepsy as a brain network disorder (Spencer, 2002; Bartolomei et al., 2008a,b, 2017; Amiri et al., 2020; Amorim-Leite et al., 2020; Gupta et al., 2020) and suggestions to not only find epileptogenic tissue but the network generating the seizures (Spencer, 2002; Prasad et al., 2003; Boling et al., 2009; Jehi, 2015). Motivation for identifying the seizure network is readily found in cases of medication-resistant epilepsy, where in current practice, removal of the seizure onset zone (SOZ) does not always control seizures (Prasad et al., 2003; Boling et al., 2009). Presently, however, the extent of structural anomalies and functional disturbances that characterize the seizure network, how these disturbances generate seizures, and which critical portions of the network need to be removed to abolish seizures, is unknown.

Studies of the seizure network using interictal EEG functional connectivity suggest brain regions in the SOZ are more strongly connected than regions not part of the SOZ (NSOZ) and possibly disconnected from the NSOZ (Warren et al., 2010; Bettus et al., 2011). Also, more connectivity alterations in NSOZ correlate with a larger epileptogenic network (Lagarde et al., 2018). Undoubtedly multiple, complex mechanisms contribute to differences in the strength of connectivity, and we believe the basis for this involves the synchrony of excitatory and inhibitory activity that could be greater in regions generating seizures than those not (Jiang et al., 2022). If this hypothesis is correct, we reasoned gamma-band connectivity might detect differences in synchrony since gamma involves coordinated synaptic activity of excitatory and inhibitory cells (Bartos et al., 2007; Buzsáki and Wang, 2012; Chen et al., 2017; Gu et al., 2021), is associated with excitatory-inhibitory balance (Gao et al., 2017), and power positively correlates with neuronal spiking rate (Mukamel et al., 2005; Manning et al., 2009). In addition, we computed theta-band connectivity because others had found differences in the theta power between mesial temporal and extratemporal lobe regions involved in generating seizures (Bettus et al., 2008).

There are a number of approaches to measure functional connectivity, including correlation (Adey et al., 1961; Alonso et al., 1996; Pfurtscheller and Andrew, 1999), phase-based methods (Lachaux et al., 1999; Mormann et al., 2000; Reijneveld et al., 2007), and information theory (Afshani et al., 2019; Ursino et al., 2020). Among these approaches is event connectivity, which combines aspects of correlation and information theory (Kheiri et al., 2013). Although not used extensively and, to our knowledge, not in patient studies of epilepsy, gamma event connectivity in rats produces stable values within behavioral states, correlates with neuronal discharges, and is sensitive to changes in excitatory and inhibitory synaptic activity (Bragin et al., 2014). Based on these data, event connectivity appears well suited for our purposes to measure functional connectivity and indirectly the synchrony of inhibitory and excitatory activity associated with the seizure network.

In the current study, we computed the strength of event connectivity in theta (4–8 Hz), low-gamma (30–55 Hz), and high-gamma (65–95 Hz) bands from interictal EEG recorded between pairs of contacts on intracerebral electrodes implanted in patients who had resective surgery or received an electrical stimulation device to control their seizures. We predicted stronger synchrony and thus event connectivity between brain regions in the SOZ than NSOZ, and larger differences in strength of connectivity between SOZ and NSOZ in seizure-free than not seizure patients, which we suspected could be due increased synchrony in some regions of NSOZ that are involved in generating seizures in not seizure-free patients.

## Materials and Methods

### Subjects and clinical recordings

All 43 subjects (26 females, 17 males) for this retrospective study were patients with medication-resistant focal seizures suspected to begin in the temporal lobe and candidates for epilepsy surgery, but required intracranial depth electrode EEG (iEEG) studies to localize the brain area of seizure onset. All patients were bilaterally implanted with seven- to nine-contact clinical depth electrodes (Ad-Tech Medical Instrument) oriented perpendicular to the lateral surface of the temporal bone and positioned to sample amygdala, entorhinal cortex, hippocampus, and parahippocampal gyrus, as well as extratemporal areas such as orbitofrontal cortex, anterior cingulate gyrus, supplementary motor areas, or parietal cortex (Table 1). Patients were recorded for 7–14 days in the epilepsy monitoring unit until a sufficient number of the patient’s habitual spontaneous seizures were captured. Depth EEG recordings were reviewed by the attending neurologist who identified the electrode contacts where seizures first appeared, which were labeled as the seizure onset zone (SOZ). All remaining contacts were considered outside or non-SOZ (NSOZ). Informed consent was obtained from each patient before the implantation of depth electrodes and participating in this research, which was approved by the Medical Institutional Review Board 3 (10-001452).

**Table 1:** Intracerebral electrodes positions in all 43 patients

	Temporal lobe										Frontal lobe						Cingulate cortex			Parietal lobe				Occipital lobe	
Patient	A	EC	MH	AH	PH	PHG	STG	TP	PT	FG	OF	SMA	FP	FO	F	SS	AC	MC	PC	IP	AP	PTB	SG	OT	O
1	RL	RL		RL		RL																			
2			RL		R		R					RL					R			R	R				
3	RL	L		L	R	L					L						R								
4	RL	RL		RL		RL					RL														
5	RL	R		RL	RL						RL														
6	RL	RL		RL							RL														
7	RL	R		RL		R	RL									R		R							
8	RL	RL		RL							RL	RL					RL								
9	L	L		RL		L		L			RL						L								
10	RL	R		RL			R				RL						R								
11	RL	RL		RL		RL					RL						RL								
12	RL	RL	RL								RL	RL					RL								
13	RL	RL		RL		RL					RL														
14	R	RL	RL								RL														
15	RL	RL	RL								RL		RL				L								
16	RL	RL		RL		RL					RL														
17	R		RL				RL									R				RL					
18	RL	RL	RL								RL														
19	R		R		R	R	R				RL														
20	RL	RL	RL			RL					RL														
21	RL	RL	RL			RL					RL														
22	L	RL				R				L														RL	
23	RL	RL	R	L		RL					RL														
24	R	RL	RL								RL	RL					R	R							
25	RL	L	RL			L					RL							L						RL	
26	R	RL	R	L		R					RL						R								
27	RL	RL	R			R					RL														
28	RL	RL	RL								RL	L					L		L						
26	R	RL	L	RL	R																			RL	
27	RL	RL	RL			RL													R					R	
31	R	RL		R		R	R				R						R								
28	L	RL	L	R		RL	L				R														
29	RL	RL		RL		RL					RL														
30	L	RL		R	L	L					L			L									L		
31	RL	RL		RL		L					L						L								
36	L		L			RL	R				RL						RL								
32	RL	RL		RL							RL						RL								
38		RL		RL		R	RL	RL	RL																
34		RL	RL				L		R										L	R					R
40	L	RL		RL			L																		
41				L			RL											R	R					R	
42	R	RL	R		R													R		R					R
43	RL	RL	RL		R						RL						RL								

TP = temporal pole, FP = frontal pole, A = amygdala, OF = orbitofrontal, EC = entorhinal cortex, F = frontal lobe, AH = anterior hippocampus, FO = frontal operculum, MH = middle hippocampus, AC = anterior cingulate, PH = posterior hippocampus, MC = middle cingulate, PHG = parahippocampal gyrus, PC = posterior cingulate, FG = fusiform gyrus, SMA = supplementary motor area, PT = posterior temporal, SS = supra-sylvian, STG = superior temporal gyrus, AP = anterior parietal lobe, IP = inferior parietal lobe, SG = supramarginal gyrus, PTB = parietal-temporal border, O = occipital lobe, OT = occipital-temporal border, R: Right, L: Left.

### Depth electrode recordings and localization

Interictal depth EEG recordings were reviewed to remove signals containing electrical noise and the remaining signals notched filtered at 60 Hz. For each patient postoperative CT scans were registered to preoperative MRI to identify electrode contacts within gray matter, and those contacts fully in white matter were excluded from the analysis as they would induce spurious connections that are a result of volume conduction. These steps yielded a total of 2055 electrode contacts without electrical noise with an average of 49 ± 16 contacts per patient. For each patient a 10- to 15-min interictal depth EEG recordings were selected using the following criteria: (1) >24 h after electrode implantation, (2) before tapering of anti-seizure medications, (3) at least 6 h before the first recorded seizure, and (4) period of quiet wakefulness with eyes open or closed. Only seizures, as an epileptiform activity, were avoided. All other interictal discharges, including epileptic spikes could have appeared in the selected data portions. Fifteen patients were recorded with a sampling frequency of 200 Hz, and 28 patients had recordings of 2 kHz. All recordings were resampled to 1 kHz using the MATLAB anti-aliasing resample function before connectivity measure was calculated. To verify sampling rate did not affect connectivity, especially with high gamma (65–95 Hz), we compared (1) the ratio of low (30–55 Hz) to high gamma power, and (2) the ratio of number of events of low to high gamma detected using the MATLAB function findpeaks between patient data with different sample rates. For each patient we calculated ratios from a randomly selected 30-s window on five channels and repeated the procedure 10 times, which generated 1400(8×5×10) datapoints for the patients sampled at 2 kHz and 750(15×5×10) datapoints for the patients sampled at 200 Hz. Results show a significant, but small effect, of sampling rate on the ratios of low to high γ power (Wilcoxon test, *p* = $7.595\times 10^{-35}$, Cohen’s *d* = 0.2001) and number of events ( $4.6258\times 10^{-28}$, Cohen’s *d* = 0.105; Extended Data Fig. 1-1*A*,*B*), suggesting the anti-aliasing filter had only a small effect on connectivity. Also, oversampling to 2 kHz produced more events needed in the peri-event histogram (see below, Connectivity metrics) but did not affect the spectral frequency components of the signal (see Extended Data Fig. 1-1*C*,*D*).

**Figure 1.**
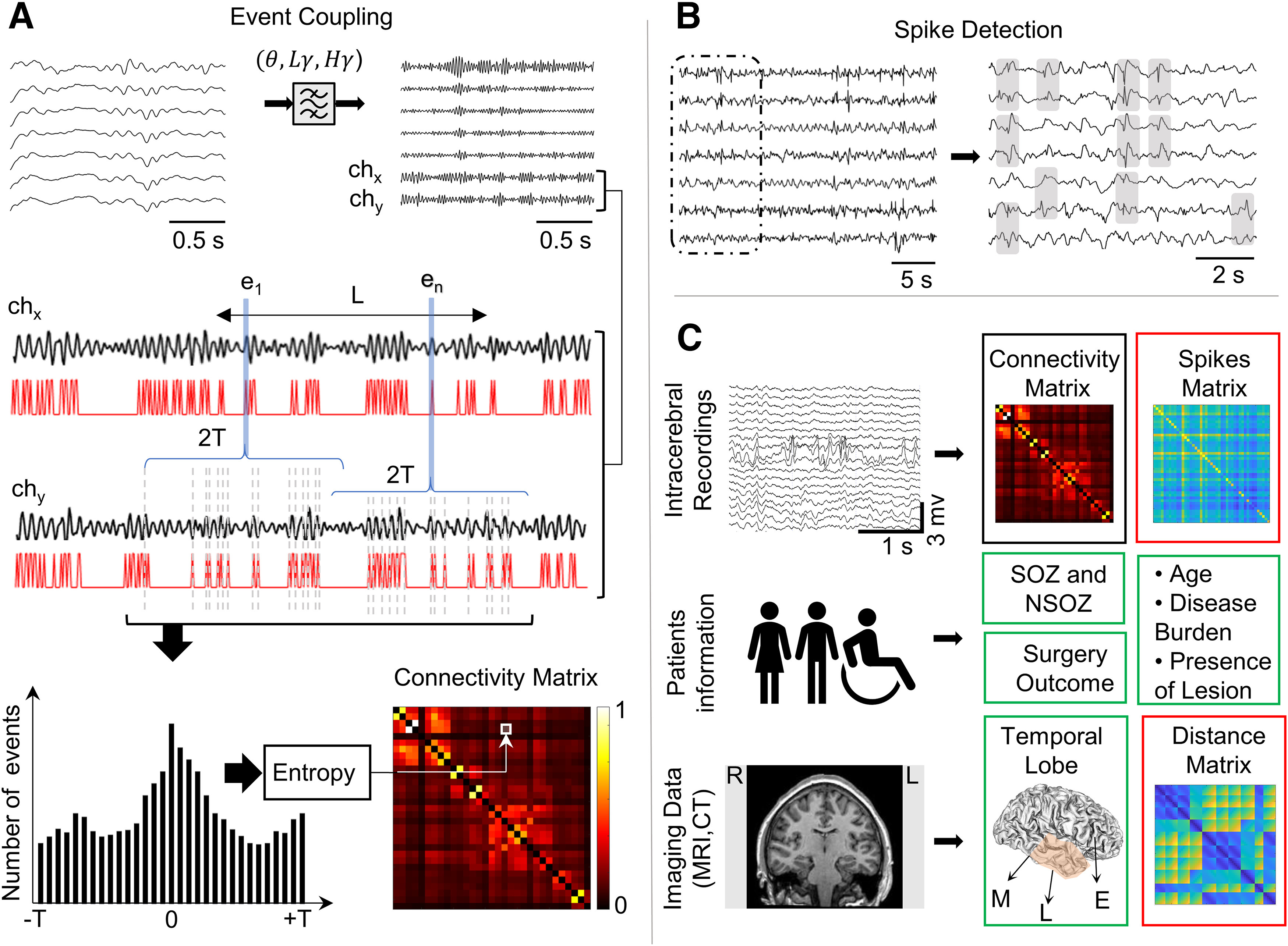
EEG analysis pipeline. ***A***, Unfiltered intracerebral EEG signals are bandpass filtered to extract spectral frequencies in theta ( θ), low gamma (Lγ), or high gamma bands ( Hγ). Functional coupling between a pair of channels (ch_x_, ch_y_) is illustrated in second row. A frequency-dependent time interval L (30 s for Hγ, 60 s for Lγ, and 5 min for θ) is chosen, and from the signals on ch_x_ and ch_y_, the local event’s amplitude maxima e_i_ (i = 1 … n) in L are detected (represented in red traces). For each e_i_ in ch_x_, the lead or lag in relation to events in ch_y_ within time interval [–T, T] is quantified in a peri-event histogram (bottom left). The distribution of the histogram is evaluated using Shannon entropy, and a low entropy value is an indication of a peak in the histogram, which represents the strength of functional coupling for every pair of channels in the connectivity matrix (bottom right). Patients with 2-kHz sampling rate (*N* = 15) and those with 200-Hz sampling rate (*N* = 28) were both used in this study, refer to Extended Data Figure 1-1 for detailed justification. ***B***, Spikes are detected from unfiltered interictal data using an automatic detector based on signal whitening. The gray boxes show the detected spikes on different channels. For every pair of contact coupling strength is computed as a rate of the sum of spikes on each channel divided by the recording duration in minutes. ***C***, Statistical model includes EEG recordings to generate functional connectivity matrix (black box) and the spikes matrix (red box), patients information and test results to assess seizure onset zone (SOZ), surgery outcome, and other measures (e.g., seizure frequency), and CT scans co-registered to MRI scans to localize electrode contacts, group contacts with respect to brain region (green box), and calculate the distance between each pair of contacts to generate distance matrices (red box). Ipsilateral and contralateral grouping was ignored (see Extended Data Fig. 1-2).

10.1523/ENEURO.0141-22.2022.f1-1 Extended Data Figure 1-1 Effect of low sampling and up-sampling on HGEC. ***A***, Ratio of low γ events to numbers of high γ events is box-plotted for patients with 2-kHz sampling (*N* = 15) and those with 200-Hz sampling rate (*N* = 28). ***B***, Same as ***A*** but for ratio of powers instead of ratio of number of events. We calculated those measures on five channels randomly selected from each patient on a randomly selected 30-s window and repeated the procedure 10 times. In total, we had 750=15×5×10 datapoints for the patients sampled at 200 Hz, and 1400=28×5×10 datapoints for the patients sampled at 2 kHz. ***C***, Two signals extracted from the right amygdala for first patient in Table 3 sampled at 200 Hz (left) are illustrated with their corresponding high γ band-filtered signals (65–95 Hz) and train of high γ events are presented underneath. The up-sampled signals (1 kHz) and train event is present to the right. ***D***, Power spectrum for the raw signal (RA2 in ***C***, blue) and for the filtered signal (orange) are presented. The power spectrum of the up-sampled signal is presented to the right. Download Figure 1-1, TIF file.

### Connectivity metrics

Connectivity measures used in previous studies are diverse. Generally, functional connectivity methods can be divided into three main categories: (1) amplitude-based measures such as different variants of the well-known amplitude correlation/coherence in time/frequency domains (Adey et al., 1961; Alonso et al., 1996; Pfurtscheller and Andrew, 1999); (2) phase-based measures where phase locking value (Lachaux et al., 1999), mean phase coherence (Mormann et al., 2000), and phase lag index (Reijneveld et al., 2007) are most frequently used; and finally; (3) connectivity originating from information theory like mutual information (Afshani et al., 2019) and transfer entropy (Ursino et al., 2020). Connectivity methods based on information theory can capture the nonlinear interactions between pairs of brain regions without a prior assumption of a predefined linear or nonlinear model that the oscillatory phase/amplitude coupling methods are usually bound to. To exploit the benefits of both correlation and information theory, we chose to use a stable connectivity measure called gamma event coupling, initially described by Bragin et al. (2014). This method is very similar to transfer entropy and mutual information where all of them use Shannon entropy to assess the strength of connectivity of a joint probability distribution but differ in the way the distribution in constructed from the available data.

The method was adapted with different windows sizes to accommodate connectivity for theta (Theta, 4–8 Hz), low gamma (LGEC, 30–55 Hz), and high gamma (HGEC, 65–95 Hz; Fig. 1*A*) event connectivity. Event connectivity was estimated based on the temporal relation between individual cycles or events of theta, low gamma, and high gamma recorded on every pair of electrodes contacts. Note that the band 55–65 Hz was omitted to reduce the chances of spurious connections resulting from 60-Hz powerline noise contamination. First, data were either down-sampled or up-sampled to 1 kHz then bandpass filtered (FIR, order) into the respective spectral frequency bands (see Fig. 1*A*). For each frequency band local amplitude maxima were detected using the “findpeaks” algorithm in the MATLAB toolbox where we used a “Threshold” parameter of value 0.1 (see Fig. 1*A*, red signal). To measure connectivity between contacts or “channels” (ch_x_ and ch_y_), peri-event histograms were used to quantify the lead or lag between each local maxima on ch_y_ and ch_x_ (e_i_ where i = 1…n, where n is total number of events in an interval of length L) within time interval [–T, T]. The values of L and T were adjusted as a function of the targeted frequency bands. According to the Nyquist rate, the highest observable frequency of events should be half the sampling rate (1000 Hz/2 = 500 Hz). A time resolution of 2 ms (1/500 Hz) can be used to distinguish two cases. As a result, we chose a bin size of 2 ms. A frequency dependent time window T was chosen as 1/fmin where fmin is the minimum frequency at which a related event might occur. In case of low gamma ( Lγ), the selected frequency band has a minimum of fmin = 30 Hz, thus T is 1/30 Hz ≈34 ms. A statistically valid histogram contains at least 30 data points per bin; therefore, 1020 (34 bins × 30 events) events need to be collected with minimum file duration of 24 s [ ≈1020 events/42.5 Hz, where 42.5 Hz = (fmin + fmax)/2]. Based on these calculations, a window length of L = 30, 60, and 300 s was used for Hγ,Lγ, and θ, respectively. This resulted in a 3D matrix of size $M\times N_{c}\times N_{c}$, where $N_{c}$ is the number of channels and M is the number of matrices corresponding to different windows. An average over all M windows was then calculated for each patient.

**Figure 2.**
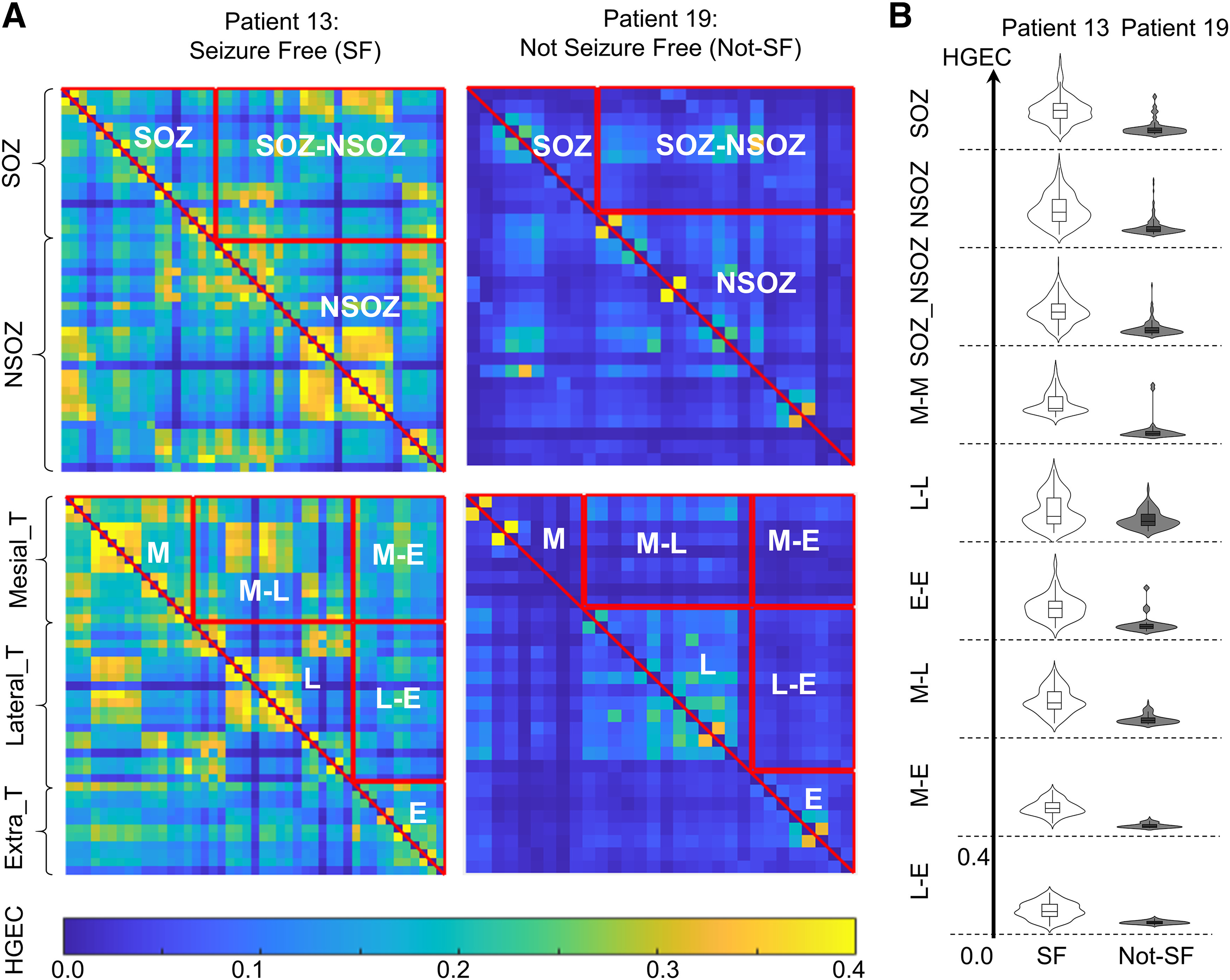
High gamma event coupling in the SOZ and different brain regions. ***A***, Examples of connectivity matrices of high gamma event coupling (HGEC) for patient 13 who was seizure-free (SF) and patient 19 who was not seizure-free (not-SF). The matrices are organized with respect to SOZ. If both electrode contacts are in SOZ, then the connectivity value is part of the SOZ, if both contacts are outside SOZ, then it is part of the NSOZ (complement), otherwise it is between the SOZ and NSOZ. The lower row illustrates HGEC organized by brain region (M: mesial temporal, L: lateral temporal, E: extratemporal). ***B***, Violin plot and box plot (inside) shows the distribution, median and interquartile range of HGEC values for patients 13 and 19 with respect to SOZ (top rows) f and brain regions (bottom rows). In most cases, HGEC is stronger in patient 13 than patient 19. Check Extended Data Figure 2-1 for GEC matrices of all patients.

10.1523/ENEURO.0141-22.2022.f1-2 Extended Data Figure 1-2 HGEC in NSOZ ipsilateral versus contralateral. Comparison between HGEC connectivity values in channels located outside the SOZ but in same hemisphere (ipsilateral) and those in the opposite hemisphere (contralateral). ***A***, Connectivity matrix where the NSOZ is organized by connectivity within the ipsilateral hemisphere (I), contralateral hemisphere (C), and between ipsilateral and contralateral hemispheres (I-C). ***B***, Boxplots illustrate the connectivity values of ipsilateral NSOZ channels and contralateral NSOZ channels. No significant difference was obtained (effect size $\eta^{2}<0.01$). Download Figure 1-2, TIF file.

When a peri-event histogram had a large peak, the two channels from the histogram were considered functionally related. Shannon entropy (S) was used to determine the peak’s power in the histogram, which is defined as:

(1) $$S=\overset{N}{\underset{i=1}{\sum }}p_{i}Ln(p_{i}),$$

where *N* is the total number of bins and $p_{i}$ is the probability of an event belonging to the *i*th bin. A lower *S* signifies stronger connectivity and a higher *S* represents weaker connectivity and a quasi-uniform distribution of events. Hence the maximum value of *S* would occur when all events have the same likelihood of occurrence ( $p_{i}=1/N$), thus $S_{max}$ is defined as:

(2) $$S_{max}=-\overset{N}{\underset{i=1}{\sum }}\frac{1}{N}Ln\left(\frac{1}{N}\right)=Ln(N).$$

The Shannon entropy value of each pair of channels ( i and j) were then normalized by subtracting it from its maximum $S_{max}$and then dividing by it by $S_{max}$ as follows:

(3) $$h_{ij}=\frac{S_{max}-S_{ij}}{S_{max}}.$$

The obtained value $h_{ij}$ represent the connectivity index (strength) between two channels ( i and j), it has a minimum value of “0” that means fully disconnected and maximum value of “1” representing a fully connected pair. Connectivity was organized into a “connectivity matrix” where the *i*th row and *j*th column of the matrix correspond to the connectivity strength between channels i and j. Note that the connectivity matrix is a symmetrical matrix, i.e., $h_{ij}=h_{ji}$.

#### Spikes coupling rate

In each patient’s recording, interictal spikes (IIS) were detected using an automatic algorithm based on whitening of the power spectrum (Roehri et al., 2016). The output of the algorithm was visually inspected to ensure correct detection of spikes (Fig. 1*B*). For a quantitative validation, we calculated the percentage of channels with top 5% spike rates from the automated spike detection and compared these channels to those labeled by the neurologist as channels with interictal discharges. Results are summarized in Table 2. Like functional coupling in the theta and gamma frequencies, the strength of IIS coupling between two channels ch_i_ and ch_j_ was computed as the sum of the total number of spikes on each channel divided by the total duration in seconds. The spikes rates were organized into matrices such that the coupling rate $r_{ij}$ found at the row i and column j represented the spikes coupling rate between channels i and j.

**Table 2:** Validating spike detector results

Patient	Spiking sites	Detector highest 5% electrodes	Percentage
1	RAH1-3, RA1-3, REC1-3, RPHG1-3, LA1-3	**RAH1, RAH 2, REC 1**	100%
4	RA1-3, REC1-3, RAH1-3, RPHG1-3, LA1-3, LEC1-3, LAH1-3, LPHG1-3	**RA1, RA2, LAH2, LAH3, LAH1**	100%
5	RAH1-3, RA1-3, RPH1-3, LAH1-3, REC1-3	**RPH3**, **RAH3**, **RA2**, ROF3	75%
7	RA5-6, REC5-6, RAH1-6, RPHG3-6, RSTGP2-3, RMC1-4, LAH1-2	RA2, **RAH1, RAH2, RPHG6, RPSTG2**, REC2, **RAH5**	70%
8	LA3-4, LAH1-2, LEC1-4, RA1-2, RAH1-2	**RAH1, LEC1,** REC1, REC2**, RAH2**	60%
11	LAH1-3, LA1-3, LEC1-3, RAH1-3, RA1 3, REC1-3	RA7, **REC3, LEC 1, REC1, LAH3**	80%
14	LEC1-3, LMH1-3	**LEC1, LEC2, LMH1**, LA1	75%
15	REC4-5, RMH1-2, LEC1-2, LMH1-2	**REC5,** LEC3, **RMH1**	66%
16	RA1-7, RAH1-4, REC1-7, RPHG4-7, ROF3-7, LA1-7, LEC1-7, LAH1-7, LPHG1-7	**REC1, REC2, LA2**	100%
17	RSTA2-3, RSTP3-4	**RSTA3,** RMH1**, RSTP3**, RIPP8, LST5	40%
18	REC1-4, RMH1-3, LA1-2, LEC1-2, LMH1-2	**REC1, REC2, RMH2**, RA2	75%
19	REC4-7, RMH4-7, RPH4-7, RSTG1-7	RA2, **RMH5,** RA1, **RMH4**	50%
20	RA1-2, REC1-2, RMH1-2, RPHG1-2, LA1-2, LEC1-2, LMH1-2, LPHG1-2,	**REC1,** RMH3, **LMH1, LEC2**	80%
21	RA1-2, RMH1-2, LEC1-3, LMH1-2, LA1-2	RA4, RMH5, **LEC1, LA1**	50%
22	LEC1-3, LA1-3, LEC4-7, LA4-7	**LEC,1, LA1,** RA1, RAH1	50%
23	RA1-3, REC1-3, RMH1-3, LA1-3, LEC1-3, LMH1-3	**LEC1,** REC4, LMH4, RMH7	25%
24	REC1-4, RMH1-2, RA1-3	**RA1, RA2**, LAH1, LA1, LAH2	40%
25	RTO5-7, RMH1-3, LA6-7, LPH5-6	**RMH2,** LPH4**, LPH5, LPH6**	75%
26	LAH1-3, LEC1-2, RMH1-2, RPHG1-3, REC1-3	**LAH1, LAH2, LAH3**	100%
27	REC1-3, RMH1-2, RPHG1-2, LMH1-2	**RMH2,** RMH4**, RPHG2, LMH1**	75%
28	LMH4-7, LEC4-7, LA4-7, LPSM4-7, LOF4-7, LAC4-7, REC4-7, RMH4-7, RA4-7	**LMH4, LMH6, LPSM5, LMH5**	100%
29	RAH1-3, RA1-3, REC1-3, RPHG1-3	LEC7, LEC6, **REC3, REC2, REC1**	60%
30	RA1-2, REC1-2, RMH1-2, RPHG1-2, LA1-2, LEC1-2, LMH1-2, LPHG1-2	**RA1, LMH1, RA2, REC1**	100%
31	RA1-7, REC1-7, RAH1-7, RPHG1-7, ROF1-3, RAF1-3, RAC5-7	**RAH1, REC1, REC2, RPHG1, RAH2**	100%
32	LEC1-3, LMH1-2, RAH1-7, REC1-7, RPHG1-7	**LEC1, LEC3, REC7, RPHG7**	100%
33	REC1-3, RAH1-3, RPHG1-3, LAH1-2, LPHGA1-2, RPHG7-8, LPHGA7-8	**REC1, REC2, RAH1, RPHG3**	100%
34	LPH1-2, LEC1-2, LA1-2	**LEC1,** LPHG7, **LEC2, LPH1**	75%
35	LAH1-2, LEC 1-2, LA 1-4, LPHG1-3	LA3, RA3, **LA2**	66%
36	LMH1-2, LPHG1-3, LA1-2, ROF4-5	**LPHG1, LPHG2, LA1**	100%
37	LEC1-2, LAH1-2, REC1-2, RAH1-2	LA1, LA2, **LAH1,** LA7	25%
38	RSTG1-4, RPT 7-8, LPT1-2, LSTG5-7, LEC5-7, LAH5-7, LMTG3-7, REC3-7	**LAH6, LAH5**, RAH6, RPHG6	50%
39	REC1-3, RMH1-3, RMNH3-7, RPNH2-6, RINH4-6, LPC 5-8	**RMH1, LPC7, RMH3, RMNH5**	100%
40	LEC1-4, LA1-4, LA5-7, LAH1-4, LPNH6-8, LSTG1-5	**LEC1, LEC2, LA1, LEC3**	100%
41	RSTGA1-4, RSTG1-4, LSTG1-4	**LSTG2,** RPST4**, LSTG1, LSTG3**	75%
42	REC1-7, RSTA1-7, RSTG1-7, RMH1-7, RIF1-7, RSO3-7, RIO3-7, RAIP3-7	RIO1, **RSTG4, RAIP7, RMH6, RSTG3**	80%
43	LEC1-3, LA1-3, RA1-3, REC1-3, LOF3-7, ROF3-7	LA5**, LOF3, RA3, RA2, LEC1**	80%

Twelve patients showed 100% correspondence in the top 5% of channels with the highest spike rates between the automated spike detector and those manually identified channels containing interictal discharges. Twenty patients showed at least 50%, 11 of which with >70% correspondence. Only four patients <40% correspondence.

R: right, L: left, TP = temporal pole, FP = frontal pole, A = amygdala, OF = orbitofrontal, EC = entorhinal cortex, F = frontal lobe, AH = anterior hippocampus, FO = frontal operculum, MH = middle hippocampus, AC = anterior cingulate, PH = posterior hippocampus, MC = middle cingulate, PHG = parahippocampal gyrus, PC = posterior cingulate, FG = fusiform gyrus, SMA = supplementary motor area, PT = posterior temporal, STG = superior temporal gyrus, AP = anterior parietal lobe, O = occipital lobe, OT = occipital-temporal border.

#### Euclidean distance connectivity

After electrodes contacts were localized. First, the anatomic image is co-registered with the CT image to mask nonbrain signals. The masked CT image is then processed (thresholded, eroded, Gaussian filtered, multiplied) to highlight electrode locations. This highlighted CT is then transformed to MNI space and loaded into iElectrodes toolbox (Blenkmann et al., 2017), where electrodes contacts were localized, labeled, and indexed. iElectrodes toolbox is a comprehensive open-source toolbox for depth and subdural grid electrode localization. The *x*, *y*, and *z* coordinates for each contact in gray matter was extracted according to the MNI system of coordinates whose origin is situated anterior commissure and has an RAS orientation. The unit of measurement was the millimeter. The Euclidean distances were then arranged into a distance matrix (Fig. 1*C*), where the distance $d_{ij}$ found on row iand column j represented the distance between channels i and j.

### Exponential model

To assess the change in connectivity strength in relation to distance we used an exponential decay model of the form:

$$s=Ae^{-\tau d},$$

where s represents the strength of the connectivity measure, A is the hypothetical strength at distance zero, d is the Euclidean distance between channels, and τ is the constant representing the rate at which the strength decays. As value of τ increases the connectivity strength weakens and reaches zero faster. For each patient, the model was fitted to the connectivity strength for each frequency band as a function of distance.

### Grouping of contacts and networks

Electrode contacts were grouped into mesial (M), lateral (L), and extratemporal lobe (E), which largely were in frontal lobe and rarely in parietal or occipital lobes. A pilot analysis showed there was no difference between ipsilateral NSOZ and contralateral NSOZ ( $P_{NSOZ-contra-inpsi}=0.086,\eta^{2}=0.000125;$ see Extended Data Fig. 1-2), and for this reason, the ipsilateral and contralateral NSOZs were combined. Brain network connectivity in relation to the SOZ was labeled as “inside” when both contacts were part of the SOZ, “outside” when both contacts were part of the NSOZ, or “between” when one contact was part of the SOZ and the other part of the NSOZ. A similar approach was adopted for network connectivity in relation to brain regions. Since all channels were labeled M, L, or E the six possible regional networks were M-M, M-L, M-E, L-L, L-E, and E-E. Initially, the mean connectivity strength for each brain region and SOZ network (i.e., SOZ, NSOZ, SOZ-NSOZ) was computed to evaluate connectivity between seizure-free and not seizure-free patients. However, we were not able assess the interactions between the brain region and SOZ. For example, if we consider one contact of a given pair, it might be in the mesial temporal region (M) and the other in the Lateral temporal region (L), i.e., part of the M-L network. At the same time, both electrodes might be in the SOZ and thus the connectivity is part of the SOZ network. This does not hold for all electrodes in the M-L network, i.e., not all contacts in the M-L network are necessary in the SOZ. Thus, calculating an average value for SOZ connectivity means ignoring the regions networks or vice-versa, and to consider the interaction between anatomic regions and zones, individual nonaveraged connectivity values were considered.

### Statistical analysis

To examine the effects of SOZ, brain region, and seizure outcome on HGEC while controlling for IIS rates and interelectrode distance, a linear mixed model was used with (1) HGEC as dependent measure; (2) SOZ, brain region, and seizure outcome as independent variables (fixed slopes); and (3) IIS and distance as covariates. The intercepts arising from different fits for each subject was set to be random. Dependent variables that could not be transformed into normal distribution were analyzed with nonparametric Wilcoxon test. The magnitude of difference was calculated as the difference between the estimated marginal means of groups. Cohen’s *d* (Cohen, 1988) was used to compute effect size for Wilcoxon test. Bonferroni was used to correct for multicomparisons. Pearson correlation was used to assess the linear relationship between interelectrode distance and event connectivity measures. All statistical tests were performed using SPSS software (IBM SPSS Statistics for Windows; IBM Corp, 2020) except for the nonparametric tests, which were conducted using the Statistics Toolbox of MATLAB software (The MathWorks, MATLAB, version 2020a).

### Code accessibility

Gamma event connectivity code is freely available at: https://github.com/MohamadShamas/GEC.git. To help in replication of the results, we provide a small dataset of five patients (one patient with 200-Hz sampling frequency and four patients with 2-kHz sampling frequency) on the same link. Instructions on how to use the code are listed in the readme.md file.

## Results

### Patient cohort

Forty-three patients (*n* = 26 females; mean age of 44.3 ± 10.6 years) with predominately temporal lobe seizures, surgical treatment, and seizure outcome were included in the study (Table 3). Results from depth electrode recording showed seizures began in unilateral or bilateral temporal lobe structures of 30 and 8 patients, respectively, temporal and ipsilateral frontal or parietal lobe in three patients, and bilateral temporal and frontal lobe in two patients. Eighteen were seizure free with an Engel score of IA or IB, and 25 continued to have clinical seizures after resective or RNS surgery (Engel Class IC to IVC; Extended Data Table 3-1) with average follow-up of 3.25 (±2.05) years. The proportion of females to males and median age at surgery was similar between seizure outcome groups, median age in seizure-free group was 52 years and in not- seizure-free group was 39 years old (nonparametric Wilcoxon test, $P_{age}$ = 0.0784). There was no significant difference in frequency of seizures or auras between the seizure-free and not seizure-free groups (Wilcoxon, $P_{Seizure\_Freq}$ = 0.434 and $P_{auras\_Freq}$= 0.832, respectively) or in the duration of epilepsy (median duration 26 vs 13 years; Wilcoxon, $P_{duration}$ = 0.0883).

**Table 3:** Patients cohort

Patients	Sex/age	Epilepsyduration	Seizurefrequency(/month)	Site(s) of SOZ	MRI	Resected area	Surgicaloutcome/follow-up	Pathology	IIS sites
1	F/38	36	6	RA, RAH, REC, RPHG	R/L HA	R AMTL	IIIB/73	HS, gliosis	RAH, RA, REC, RPHG, LA
2	F/17	8	90	RIP, RAP, RMH	Normal	R parietotemporal neocortex	IIC/126	Subcortical WM ectopic neurons	NA
3	F/42	30	20	LA, LEC, LAH	L HA	L AMTL	IB/51	FCD Ia	NA
4	F/39	32	5	RAH, RPHG, RA, REC	R/L HA	R AMTL	IA/43	Gliosis	RA, REC, RAH, RPHG, LA, LEC, LAH, LPHG
5	F/28	20	2	RA, RAH, REC, RPHG	Normal	R AMTL, temporal neocortical	IVB/72	Subcortical WM ectopic neurons	RAH, RA, RPH, LAH, REC
6	F/30	29	28	LA, LAH	L HA	VNS	IA/12	NA	NA
7	M/21	9	4	REC, RAH, RPHG	R FCD, PNH	R AMTL, temporooccipital	IIIA/84	FCD Ic, IIa	RA, REC, RAH, RPHG, RSTP, RMC, LAH
8	F/25	20	27	RAH, RA, REC	R/L HA	R AMTL	IB/60	None	LA, LAH, LEC, RA, RAH
9	M/42	22	16	LEC, LPHG, LA, LAH, RAH	R/L hippocampal hyperintensity	L AMTL	II/36	None	NA
10	F/48	32	9	RAH, RA	Normal	R AMTL	IIIC/42	HS	NA
11	M/40	5	1	LA, LEC, LAH	L caudate nucleus atrophy	L AMTL	IA/24	None	LAH, LA, LEC, RAH, RA, REC
12	F/20	9	12	LA, LEC	Normal	L AMTL	IIB/51	FCD IIa	NA
13	F/46	46	6	LA, LEC, LAH	L HA	L AMTL	IB/9	HS	NA
14	F/53	51	12	LEC, LMH, LA	L hippocampal hyperintensity	L AMTL	IA/86	None	LEC, LMH
15	M/45	5	8	LEC, LMH	L HA	L AMTL	IA/58	None	REC, RMH, LEC, LMH
16	M/29	8	13	RA, REC, RAH, RPHG, ROFLA, LAH	Normal	RNS anterior hippocampus	IVB/25	NA	RA, RAH, REC, RPHG, ROF, LA, LEC, LAH, LPHG,
17	F/50	24	2	RSTA, RSTP	R perisylvian polymicrogyria	R temporoparietal neocortex, STG	IB/2	Gliosis	RSTA, RSTP
18	F/49	19	3	RA, REC, RMH, LA, LEC, LMH	Normal	R AMTL	IIA/61	FCD Ic	REC, RMH, LA, LEC, LMH
19	F/41	12	30	REC, RMH, RPHG, RSTG	Normal	R AMTL, R lateral TL	IIA/17	HS, gliosis	REC, RMH, RPH, RSTG
20	M/49	31	20	RA, REC, RMH, RPHG	Normal	R AMTL	IA/1.5	FCD Ic, gliosis	RA, REC, RMH, RPHG, LA, LEC, LMH, LPHG
21	F/35	30	110	LEC, LA, RA	L HA	VNS	IA/10	NA	RA, RMH, LEC, LMH, LA
22	M/56	20	2	LA, LEC	L posterior comm. artery infarct	L AMTL	IIB/27	Subcortical WM ectopic neurons	LEC, LA
23	F/40	12	4	RA, REC, RMH, RPHG	R FCD temporal pole	R AMTL	IB/45	FCD IIb, gliosis	RA, REC, RMH, LA, LEC, LMH
24	F/34	22	8	REC, RMH	Normal	R AMTL	IVC/48	Gliosis	REC, RMH, RA
25	F/27	13	8	LEC, LTO, LPH, REC, RMH	PVH, polymicrogyria	DBS	IVB/9	NA	RTO, RMH, LA, LPHG
26	M/35	16	170	REC, RMH, RPHG	Normal	Entorhinal cortex replace RNS	IIIA/29	NA	LAH, LEC, RMH, RPHG, REC
27	F/27	7	4	REC, RMH, RPHG, RA	Normal	RNS MTLE	IIIA/74	NA	REC, RMH, RPHG, LMH
28	M/26	18	4	LEC, LA, LMH, LOF, LAC, RA, REC, RMH, ROF	Encephalomalacia L lateral superior TL	TL ATL sparing mesial structures, RNS L hippocampal-LOF	IIB/20	Gliosis, heterotopia WM	LMH, LEC, LA, LPSM, LOF, LAC, REC, RMH, RA
29	F/34	20	8	RAH, RA, REC	R/L PNH	RNS RAH and REC	IVB/45	NA	RAH, RA, REC, RPHG
30	M/27	9	1	RA, REC, RPHG, LA, LEC, LMH, LPHG	R HA	RNS L/R EC	IIIA/38	NA	RA, REC, RMH, RPHG, LA, LEC, LMH, LPHG
31	F/30	15	15	RA, RPHG	ROF atrophy	R lesionectomy	IA/34	Gliosis	RA, REC, RAH, RPHG, ROF, RAF, RAC
32	F/21	4	2	LEC, LA, LMH, LPHG	L temporal pole encephalocele	L AMTL	IB/35	HS, gliosis	LEC, LMH, RAH, REC, RPHG
33	M/51	23	4	LEC, LAH, LPHG, REC, RPHG	R HA, L FCD	RNS L/R medial TL	IB/24	NA	REC, RAH, RPHG, LAH, LPHG
34	M/58	8	1	LPH, LEC, LA, RAH, REC	L HA	L AMTL	IIIA/63	HS, gliosis	LPH, LEC, LA
35	F/49	13	3	LA, LAH, LEC, LPHG	L hippocampal hyperintensity	RNS L medial TL and L EC	IIB/28	NA	LAH, LEC, LA, LPHG
36	F/43	27	3	LOF, LMH, LPHG	FCD RT pole	R AMTL, RNS L MTL–R Middle OF	IID/36	HS	LMH, LPHG, LA, ROF
37	M/69	5	0.5	LEC, LAH	L HA	L amygdalo-hippocampectomy w/Visualase	IIIA/55	NA	LEC, LAH, REC, RAH
38	M/50	38	90	LEC, LMH, LMTG, LAH, RSTG, RTP, RPT, LPT, LAH, REC	Hyperintensity L post-TL	VNS	IVB/31	NA	RSTG, RPT, LPT, LSTG, LEC, LAH, LMTG, REC
39	F/44	9	120	REC, RMH	R/L PNH	R AMTL	IIIA/59	None	REC, RMH, RMNH, RPNH, RINH, LPC
40	F/34	8	5	LEC, LA, LAH, LSTG, LPNH, LMNH, LANH	L PVH, hypothalamic hamartoma	RNS LEC-LPNH	IIB/38	NA	LEC, LA, LAH, LPNH, LSTG
41	M/35	25	0	RASTG, RPSTG, LSTG	Hyperintensity RT pole, PVH	R AMTL, TL R superior, middle, inferior temporal extended	IV/33	Gliosis	RSTGA, RSTG, LSTG
42	M/25	15	8	RMH, RSTG, RSTA, RA, REC	Atrophy R hemisphere Vascular new infarct R post-TL	R ML TL/TL R TPO, RNS RSTG, RO	IVC/12	Gliosis, CD	REC, RSTA, RSTG, RMH, RIF, RSO, RIO, RAIP
43	M/28	25	60	LA, LEC, LMH, RA, REC, RMH	L MTS hyperintensity amygdala R>L, L ant TL	RNS L entorhinal, L anterior insula	IIIA/33	NA	LEC, LA, RA, REC, LOF, ROF

R = right, L = left, A = amygdala, AH = anterior hippocampus, MH = middle hippocampus, PH = posterior hippocampus, EC = entorhinal cortex, PHG = parahippocampal gyrus, OF = orbitofrontal cortex, FA = anterior frontal, STG/A/P = superior temporal gyrus/anterior/posterior, AMTL = anteromesial temporal lobectomy, RNS = responsive neurostimulation, NA = not available, FCD = focal cortical dysplasia, HA = hippocampal atrophy, HS = hippocampal sclerosis, PNH = periventricular nodular heterotopia, TS = tuberous sclerosis. See Extended Data Table 3-1 that shows examples for resection or RNS therapy in the SOZ.

### Connectivity in relation to SOZ, brain region, and seizure outcome

For each patient we constructed connectivity matrices computed from a 10- to 15-min period of interictal depth EEG (see Materials and Methods). Inspection of the matrices, like the example of HGEC illustrated in Figure 2*A*, revealed stronger connectivity in seizure-free than not seizure-free patients (see Extended Data Fig. 2-1). Arranging the electrodes in relation to the SOZ (Fig. 2*A*, top row) or brain region (Fig. 2*A*, bottom row) also indicated differences in connectivity in many, but not all, patients (Fig. 2*B*). To verify these observations, we used linear mixed model analysis to evaluate the effect seizure outcome as well as SOZ and brain region on Theta, LGEC, and HGEC. We included the rate of interictal spikes and intercontact distance as covariates in the model since each of these could affect connectivity (Ren et al., 2015; Lagarde et al., 2018).

10.1523/ENEURO.0141-22.2022.f2-1 Extended Data Figure 2-1 High γ events connectivity (HGEC) matrices for all seizure-free patients (green) and not seizure-free patients (red) are presented. The matrices are organized by connectivity within the seizure onset zone (SOZ; upper triangle), within the seizure onset zone complement (NSOZ; lower triangle) and between the SOZ and NSOZ (rectangle) networks. Download Figure 2-1, TIF file.

**Figure 3.**
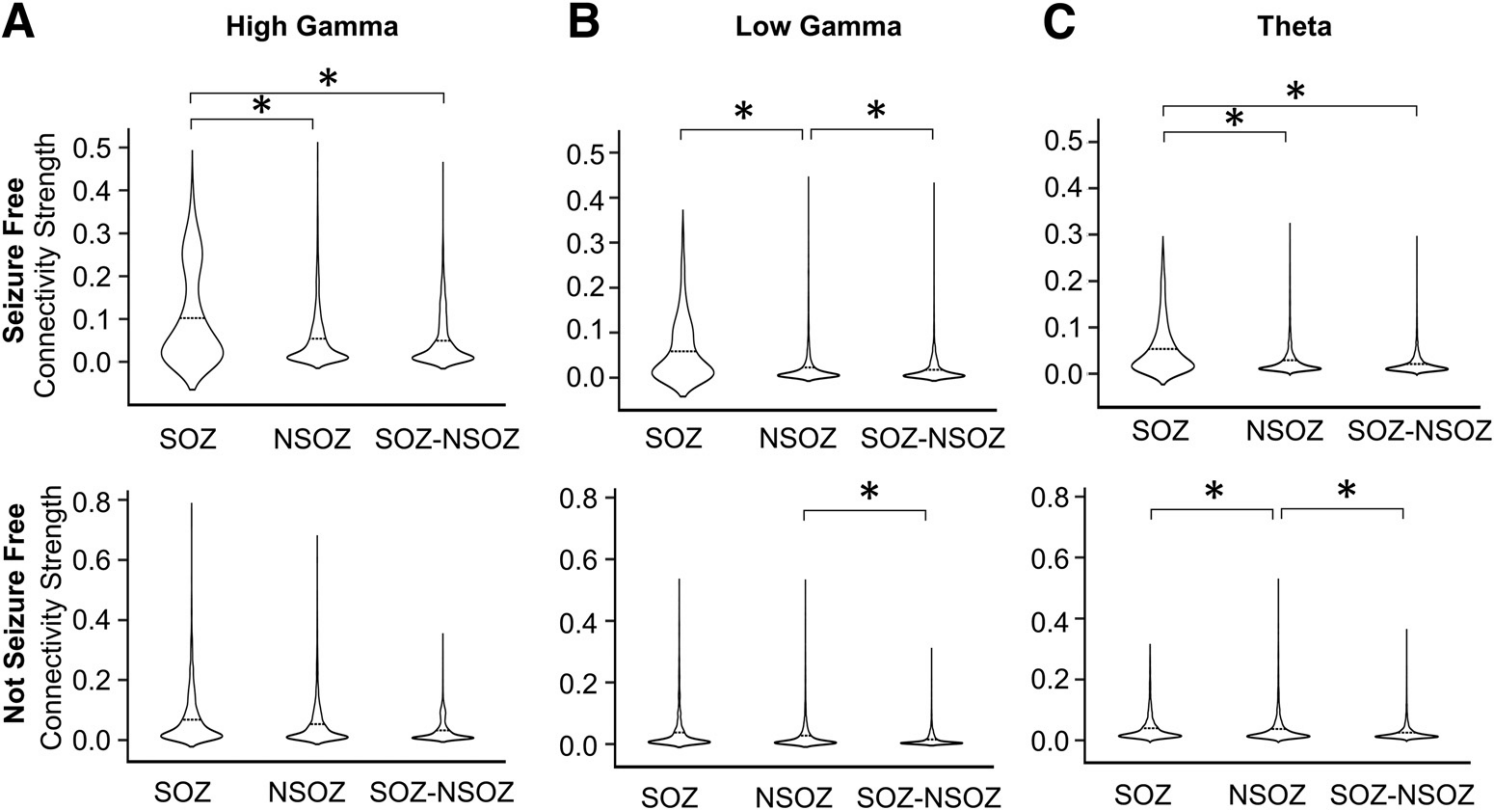
Connectivity strength in relation to seizure outcome and SOZ interaction. ***A*–*C***, Violin plots that show HGEC, LGEC, and TEC in relation to SOZ and seizure outcome (SF upper row, NSF lower row). The significant differences (*p* < 0.05) are marked by asterisks (*). Results for level 1 interactions between connectivity and either zones or outcome are depicted in Extended Data Figure 3-1.

**Figure 4.**
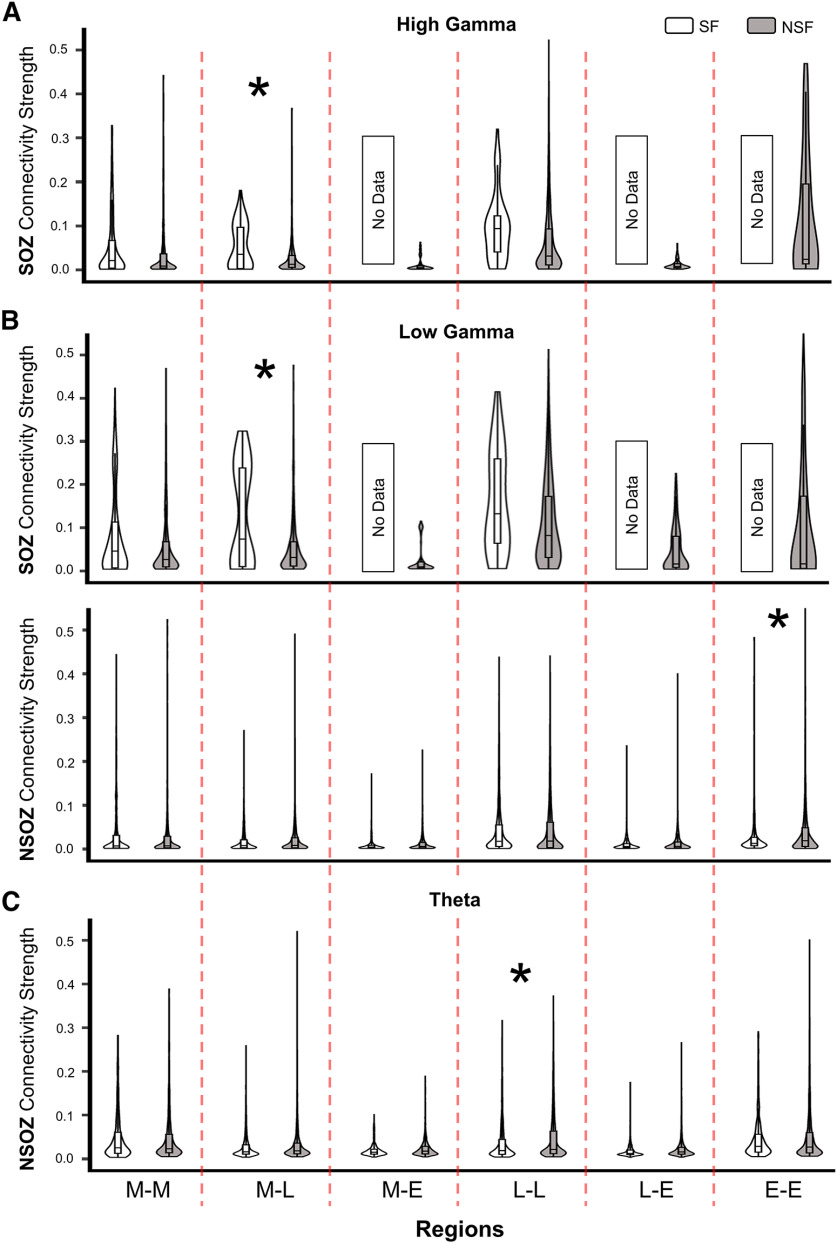
Connectivity strength in relation to seizure outcome, brain Regions and SOZ. ***A***, Violin plots show HGEC values in relation to SOZ and brain region (columns; abbreviations same as Fig. 2) for all patients. ***B***, Violin plots show LGEC values in relation to SOZ (upper row), NSOZ (lower row), and brain regions (columns) for all patients. ***C***, Violin plots show ThEC values in relation to NSOZ and brain regions (columns) for all patients. Seizure-free patients were shaded white and not seizure-free outcome were shaded black. The significant differences (*p* < 0.05) are marked by asterisks (*).

Results from the linear mixed model found seizure outcome did not have a significant effect on connectivity nor did SOZ, except on HGEC, and brain region did have a significant effect on HGEC, LGEC, and Theta (see Table 4). No significant differences were obtained when comparing different zones and seizure outcomes for all three frequency bands (see Extended Data Fig. 3-1*A*,*B*). Delving into the model’s results (i.e., interactions), results show stronger HGEC and LGEC in the SOZ than NSOZ of seizure-free patients but no difference in not seizure-free patients (see Fig. 3). Furthermore, seizure-free patients had stronger HGEC and LGEC in the SOZ between mesial and lateral temporal lobe than not seizure patients (see Fig. 4*A*,*B*). By contrast, seizure-free patients had weaker LGEC in the extratemporal NSOZ than not seizure patients (see Fig. 4*B*). Also, there was weaker Theta in SOZ than NSOZ of seizure-free and not seizure-free patients. Lastly, seizure-free patients had weaker Theta in lateral temporal lobe NSOZ than not seizure-free patients (see Fig. 4*C*). These results derive from a seizure-free group that included patients without and with aura (i.e., Engel IA and IB outcomes). When the same analysis was performed with a seizure-free cohort consisting of Engel IA only (*n* = 8 patients) there was no difference in connectivity between seizure-free and not seizure patients.

**Table 4:** Statistical table

Hypothesis	*p*-value	*F* value $\mu_{1}-\mu_{2}$ or β	Nb samples
Zone significant predictor of HGEC	**0.000497**	7.60	52,203
Zone significant predictor of LGEC	0.130	2.041	52,203
Zone significant predictor of ThEC	**0.057**	2.860	52,203
Regions significant predictor of HGEC	**2.14*e*^–191^**	180	52,203
Regions significant predictor of LGEC	**4.85^–25^**	241	52,203
Regions significant predictor of ThEC	**4.31*e*^–105^**	99.7	52,203
SF patients significantly different from NSF in HGEC	0.206	1.65	52,203
SF patients significantly different from NSF in LGEC	0.910	0.013	52,203
SF patients significantly different from NSF in ThEC	0.205	1.66	52,203
Seizure outcome significant predictor of HGEC	0.162	2.02	52,203
Seizure outcome significant predictor of LGEC	0.539	0.384	52,203
Seizure outcome significant predictor of ThEC	0.520	0.421	52,203
SOZ HGEC > NSOZ HGEC in SF patients	**1.10*e*^–9^**	18.8 ( $\mu_{1}-\mu_{2}$ = 0.0165)	401
SOZ LGEC > NSOZ LGEC in SF patients	**0.0217**	8.23 ( $\mu_{1}-\mu_{2}$ **=** 0.00412)	401
SOZ ThEC < NSOZ ThEC in SF patients	**1.12*e*^–6^**	12.1 ( $\mu_{1}-\mu_{2}$ **=** –0.00761)	2785
SOZ ThEC < NSOZ ThEC in NSF patients	**0.00855**	14.9 ( $\mu_{1}-\mu_{2}$ **=** –0.00245)	2785
In SOZ, M-L, HGEC SF > HGEC NSF	**0.00114**	11.8 ( $\mu_{1}-\mu_{2}$ **=** 0.001143)	996
In SOZ, M-L, LGEC SF > LGEC NSF	**0.00205**	9.94 ( $\mu_{1}-\mu_{2}$ **=** 0.016924)	996
In NSOZ, E-E, LGEC SF < LGEC NSF	**0.0089**	7.46 ( $\mu_{1}-\mu_{2}$ **=** –0.011616)	5069
In NSOZ, L-L, ThEC SF < ThEC NSF	**0.0111**	7.03 ( $\mu_{1}-\mu_{2}$ **=** –0.010777)	6106
Spikes significant predictor of HGEC	**1.22*e*^–100^**	455 ( β **=** 0.00410)	52,203
Spikes significant predictor of LGEC	**7.71*e*^–106^**	479 ( β **=** 0.00279)	52,203
Spikes significant predictor of ThEC	**1.54*e*^–50^**	233 ( β **=** 0.00165)	52,203
Distance significant predictor of HGEC	**7.32*e*^–110^**	5504 ( β **=** 0.900)	52,203
Distance significant predictor of LGEC	**1.40*e*^–121^**	6401 ( β **=** 0.868)	52,203
Distance significant predictor of ThEC	**2.12*e*^–87^**	3804 ( β **=** 0.717)	52,203

Significant values are shown in bold.

10.1523/ENEURO.0141-22.2022.f3-1 Extended Data Figure 3-1 Connectivity strength in relation to seizure outcome or SOZ. ***A***, Violin plots that show HGEC, LGEC, and ThEC in relation to seizure outcome where SF patients are shaded in white and NSF are shaded in black. ***B***, Violin plots that show HGEC, LGEC, and ThEC in relation to SOZ. Download Figure 3-1, TIF file.

#### Interictal spikes and connectivity

**Figure 5.**
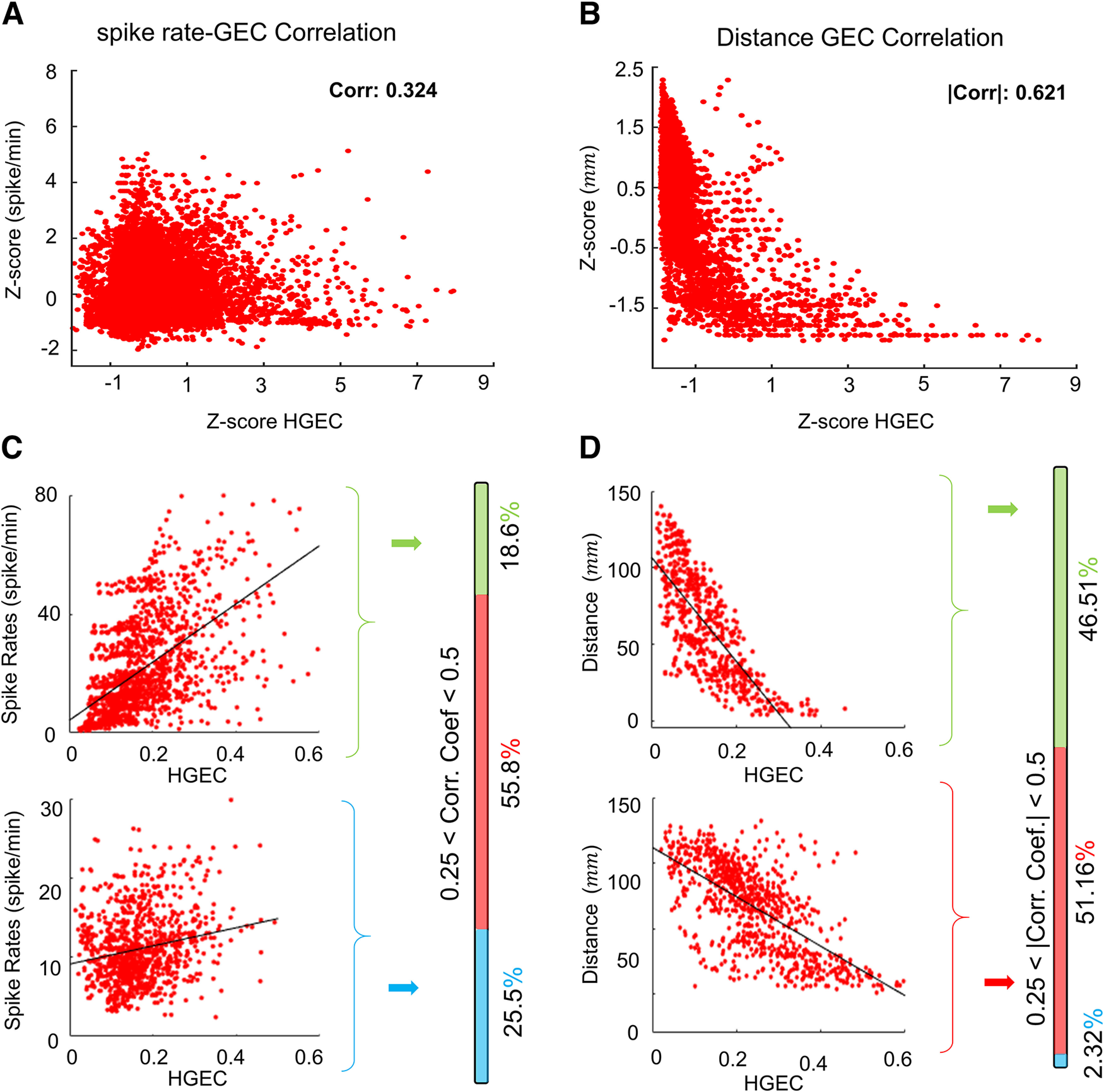
Correlation between HGEC and interictal spike rate and electrode distance. ***A***, ***B***, Scatter plots illustrating HGEC in relation to spike rate (***A***) and electrode contact distance (***B***). Values are represented as normalize *z* scores. ***C***, Specific examples of high (top) and low (bottom) correlation between spike rate and HGEC. The vertical bar to the right shows the percentage correlation coefficients for all patients. High *r* > 0.5 (shaded green), medium 0.25 < *r* < 0.5 (red), and low correlation *r* < 0.25 (blue). In most patients, the correlation between spike rate and HGEC was low. ***D***, Same as panel ***C*** but correlation with electrode distance. In most patients, there was a high correlation between electrode distance and HGEC, i.e., as electrode distance decreases, HGEC increases. All correlations shown had a *p* < 0.0001.

Previous studies found interictal spikes could affect connectivity, especially in the gamma band (Ren et al., 2015; Lagarde et al., 2018), and for this reason we included the rate of spikes in the model. The current analysis found a significant, albeit small, effect of interictal spikes on the strength of HGEC, LGEC, and ThEC (see Table 4). Consistent with the small estimated coefficients, overall analysis found a higher rate of spikes was weakly to moderately correlated with HGEC, LGEC, and Theta (*r* = 0.19, 0.29, and 0.27, respectively; see Fig. 5*A* for example of HGEC). At the level of the individual patient, 4 of the 43 patients had a strong correlation between interictal spikes and HGEC (*r* > 0.5; Fig. 5*C*, top scatterplot), but in all others, it was moderate (*r* < 0.5) or weak (*r* < 0.25; Fig. 5*C*, bottom scatterplot). The modest correlation between connectivity and spikes was unexpected and could be because of a limited sample of spikes in short duration recordings. However, there was not a significant correlation between individual *r* values of HGEC and spike rates (*r* = 0.315, *p* = 0.0576), suggesting a limited sampling of spikes alone can explain the modest correlation. Similar results were found with LGEC and Theta.

### Interelectrode distance and connectivity

The distance between electrode contacts could affect connectivity strength, i.e., shorter distances correspond with stronger connectivity (Lagarde et al., 2018). The statistical model found interelectrode distance had a significant large effect on the strength of HGEC, LGEC, and ThEC (see Table 4). Overall, shorter distances between contacts correlated with stronger HGEC, LGEC, and Theta (Fig. 4*B*, *r* = 0.45, 0.69, and 0.68 for HG, LG, and theta, respectively). In 42 out of 43 patients, there was a strong correlation between interelectrode distance and strength of connectivity (see example HGEC in Fig. 5*D*, top scatterplot), which is consistent with large estimation coefficients, and only one patient with a weak correlation (Fig. 5*D*, bottom scatterplot).

**Figure 6.**
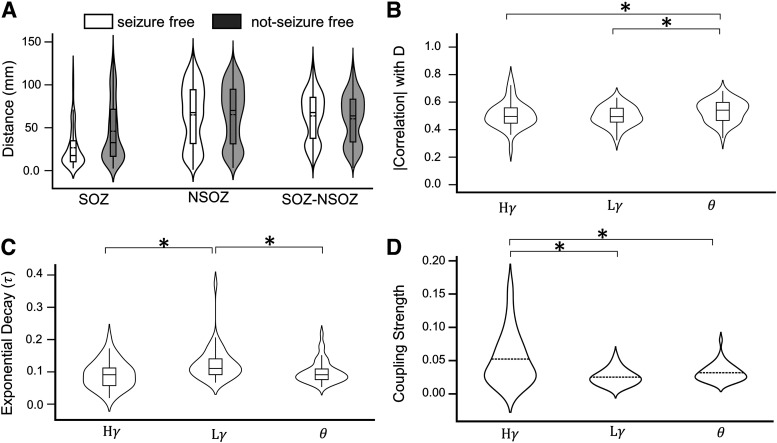
Comparing event connectivity in three frequency bands. ***A***, Violin plots show median Euclidean distance between pairs of contacts in relation to SOZ (abbreviation same as Fig. 2) and seizure outcome for all patients (abbreviation and shading same as Fig. 4). ***B***, Violin and box plots of correlation coefficient between electrode distance and strength of functional coupling in high gamma (Hγ), low gamma (Lγ), and theta (θ) frequency bands in all zones and regions. Note that each patient has one correlation value, i.e., the violin plots are for 43 points each. ***C***, The decay constant ( τ) of the exponential decay model [EC = A*exp(- τ*d)] relating the variation of event coupling strength (EC) of different frequency bands ( Hγ, Lγ, θ) with the distance (d) between channels is illustrated in form of violin plots each representing 43 patients. See Extended Data Figure 6-1, which illustrates the difference between slow and fast decays. ***D***, Coupling strength for Hγ, Lγ, and θ are compared; *p* < 0.05 denoted by asterisks (*).

Next, we analyzed interelectrode distance in relation to the SOZ and seizure outcome. Results show distances were shorter between electrodes in the SOZ (median distance ∼30mm) than those in the NSOZ (median distance ∼68mm) and between the SOZ and NSOZ (median distance ∼67mm; Fig. 6*A*). Interelectrode distances in the SOZ ( $P_{dist-soz}$ = $3.11e^{-29},\eta^{2}=0.563$) and NSOZ ( $P_{dist-Nsoz}$ = 0.00612, $\eta^{2}=0.0321$) were shorter in seizure-free than not seizure-free patients, but no differences were seen in distances between SOZ and NSOZ ( $P_{dist-soz-Nsoz}$ = 0.215; Fig. 6*A*).

10.1523/ENEURO.0141-22.2022.f6-1 Extended Data Figure 6-1 The exponential relationship between HEC and Euclidian distance is plotted slow decaying HGEC (***A***, patient 3) and fast decaying HGEC (***B***, patient 24). Download Figure 6-1, TIF file.

### Connectivity in relation severity and duration of epilepsy

**Figure 7.**
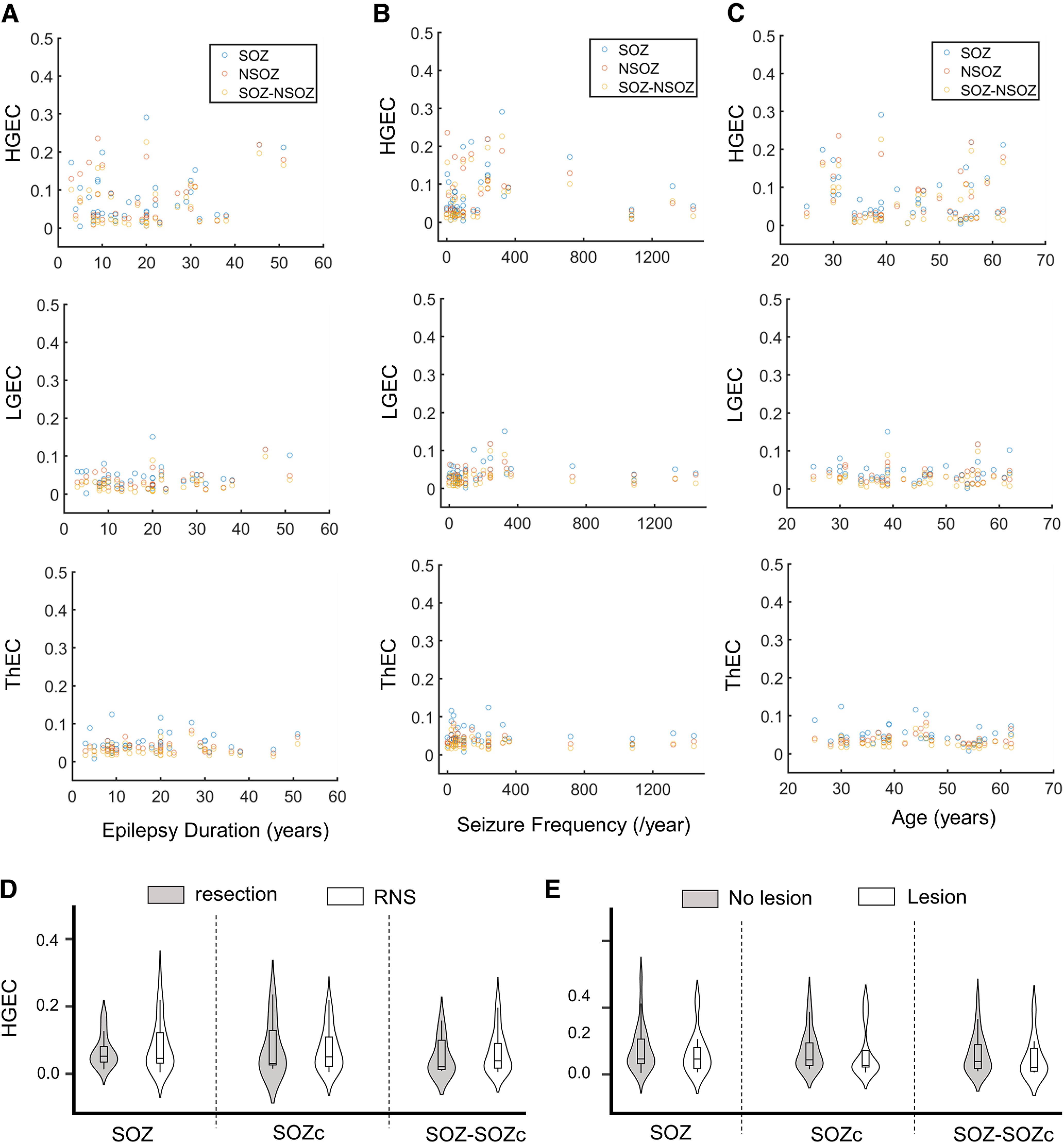
Average high gamma event connectivity (HGEC) as a function of (***A***) epilepsy duration, (***B***) seizure frequency, (***C***) patients age, (***D***) type of surgery, and (***E***) presence of an MRI lesion. Extended Data Figure 7-1 gives examples of different types of MRI abnormalities.

Difference in history or severity of epilepsy could affect connectivity; thus, we performed correlation analysis between strength of connectivity and measures of epilepsy severity and burden. Analysis found no correlation between strength of connectivity and duration of epilepsy ( $P_{dur-gec}$ = 0.15, 0.028, 0.58), seizure frequency ( $P_{seizureFreq-gec}$ = 0.59, 0.99, 0.47), age of epilepsy onset ( $P_{onset-gec}$ = 0.81, 0.84, 0.25), or burden of disease, i.e., seizure frequency/year X duration of epilepsy in years ( $P_{burden-gec}$ = 0.71, 0.37, 0.72; Fig. 7). Also, there were no differences in the strength of connectivity between patients who received a resection and those who received an RNS, or between patients with MRI lesion and those without a lesion (see Extended Data Fig. 7-1).

10.1523/ENEURO.0141-22.2022.f7-1 Extended Data Figure 7-1 Representative MRI from three patients in this study illustrating the different types of MRI pathology found in these cases that required invasive EEG. Download Figure 7-1, TIF file.

10.1523/ENEURO.0141-22.2022.fT3-1 Extended Data Figure T3-1 Resection or RNS therapy in the SOZ. ***A***, Resection of tissue corresponding to SOZ in patient 24. Postimplant CT (left, axial) registered with postsurgical MRI in coronal (middle) and axial planes (right). Red dots denote contacts of depth electrode with distal contacts positioned in right entorhinal cortex. Area outlined in white indicates the margins of resection in the plane of view. ***B***, Same as panel ***A***, but patient 39 and yellow dots denote contacts of depth electrode positioned to sample right middle hippocampus. ***C***, RNS therapy of the left mesial temporal lobe SOZ, including entorhinal cortex, in patient 35. Full-head model illustrates trajectories of two RNS probes (magenta lines) with one entry (E) from occipital cortex with contacts (magenta dots) positioned in left amygdala, hippocampus, and parahippocampal gyrus, and the other E from lateral aspect of temporal cortex with contacts in and adjacent to entorhinal cortex. Yellow dots denote depth electrode contacts of the left SOZ involving amygdala, entorhinal cortex, middle hippocampus, and parahippocampal gyrus. Sagittal view (top), clockwise-rotated posterolateral view (middle), and axial view (bottom). A = anterior, P = posterior, D = dorsal, V = ventral, L = left, and R = right. Download Figure T3-1, TIF file.

### Comparison of functional connectivity in three frequency bands

HGEC was strongest in the SOZ than LGEC ( $P_{SOZ-HGEC-LGEC}$ = 6.53 $e^{-4}$; Cohen’s *d* = 0.911) and Theta ( $P_{SOZ-HGEC-TEC}$ = 0.0480, Cohen’s *d* = 0.682; Fig. 6*D*). The correlation between interelectrode distance and Theta was stronger than the correlation between distance and LGEC ( $P_{dist-Theta-LGEC}$ = 0.0432, Cohen’s *d* = 0.334) or HGEC ( $P_{dist-HGEC-LGEC}$ = 0.0479, Cohen’s *d* = 0.219; Fig. 6*B*). An exponential model could best describe the relationship between interelectrode distance and strength of connectivity with LGEC having the faster exponential decay (median τ=0.109) than HGEC ( τ=0.0898, *p* = 0.00344, Cohen’s *d* = 0.643) and Theta ( τ=0.0901, *p* = 0.0051, Cohen’s *d* = 0.518; Fig. 6*C*). Some examples of the exponential fit are illustrated in Extended Data Figure 6-1.

## Discussion

The main findings in this study are (1) stronger HGEC and LGEC in SOZ than NSOZ of seizure-free patients; (2) stronger HGEC and LGEC between mesial and lateral temporal SOZ in seizure-free than not seizure-free patients; and (3) stronger LGEC and ThEC in extratemporal and lateral temporal NSOZ of not seizure-free than seizure-free patients. These results were unrelated to interictal spikes, clinical features of epilepsy, or MRI abnormality but were affected by interelectrode distance, which was adjusted for in the analysis. These relative differences in interictal event connectivity could indicate abnormal synchrony within and beyond the SOZ that contributes to seizure recurrence.

### Differential event connectivity with respect to SOZ and NSOZ

Studies of functional connectivity in epilepsy commonly use linear or nonlinear correlation to assess the dependency between bandpass filtered EEG signals recorded from pairs of scalp or intracranial electrodes. Several studies found stronger interictal functional connectivity in the mesial temporal or extratemporal lobe SOZ than NSOZ (Bettus et al., 2008; Bartolomei et al., 2013; Lagarde et al., 2018). Stronger connectivity was found in conventional EEG frequency bands, including gamma, which is consistent with evidence of increased gamma power in the SOZ (Worrell et al., 2004; Medvedev et al., 2011; Cimbalnik et al., 2018; Zweiphenning et al., 2019). In the current study, we computed a form of connectivity using peri-event time histograms to quantify the correlation between local maxima of individual events recorded from pairs of depth electrode contacts; a method previously used to assess event connectivity in rats (Kheiri et al., 2013; Bragin et al., 2014). With this approach we, too, found stronger LGEC and HGEC in the SOZ than NSOZ, chiefly between the mesial and lateral temporal SOZ in seizure-free patients. Furthermore, we found stronger ThEC in NSOZ than SOZ, especially in lateral temporal lobe of not seizure-free than seizure-free patients, which could be related to the reduced theta power in mesial temporal than extratemporal lobe SOZ (Bettus et al., 2008). Differences in event connectivity associated with lateral temporal lobe found in our analysis are consistent with this region’s involvement in some forms of temporal lobe epilepsy, especially those where the SOZ includes entorhinal cortex and MRI is normal or contains a lesion other than hippocampal sclerosis (Bartolomei et al., 2010), which characterizes many of the patients in the current study. To better understand the implications of these results to the seizure network and seizure outcome, it would be helpful to first explain what we believe event connectivity represents, which we discuss in the following paragraph.

### What could event connectivity represent?

Most brain rhythms like theta-band and gamma-band activity involve inhibition that can coordinate regular fluctuations in neuronal excitability, which generates coherent extracellular current flows measured in the EEG (Buzsáki and Watson, 2012). Gamma oscillations, for example, involve coordinated activity between inhibitory and excitatory cells (Buzsáki and Wang, 2012), but if there is inhibitory dysfunction, then there is greater excitatory asynchrony and increased gamma-band fluctuations (Yizhar et al., 2011; Cho et al., 2015). In the current study, it is likely LGEC and HGEC chiefly represent spontaneous gamma-band fluctuations in multiunit activity, which was shown in rats (Bragin et al., 2014) and suggested to occur in humans (Burke et al., 2015). Regarding theta, which can be recorded in human mesial temporal lobe and neocortex (Kahana et al., 2001), it is possible that ThEC could correspond with coordinated inhibitory and excitatory activity like LGEC and HGEC, but it involves a larger volume of tissue and/or greater spatial distribution of sources. Thus, we propose, in the epileptic brain, the strength of event connectivity corresponds with synchrony of inhibitory and excitatory activity such that relatively stronger event connectivity is associated with stronger synchrony and weaker event connectivity is associated with weaker or asynchronous inhibitory and excitatory activity.

### Event connectivity and seizure recurrence

**Figure 8.**
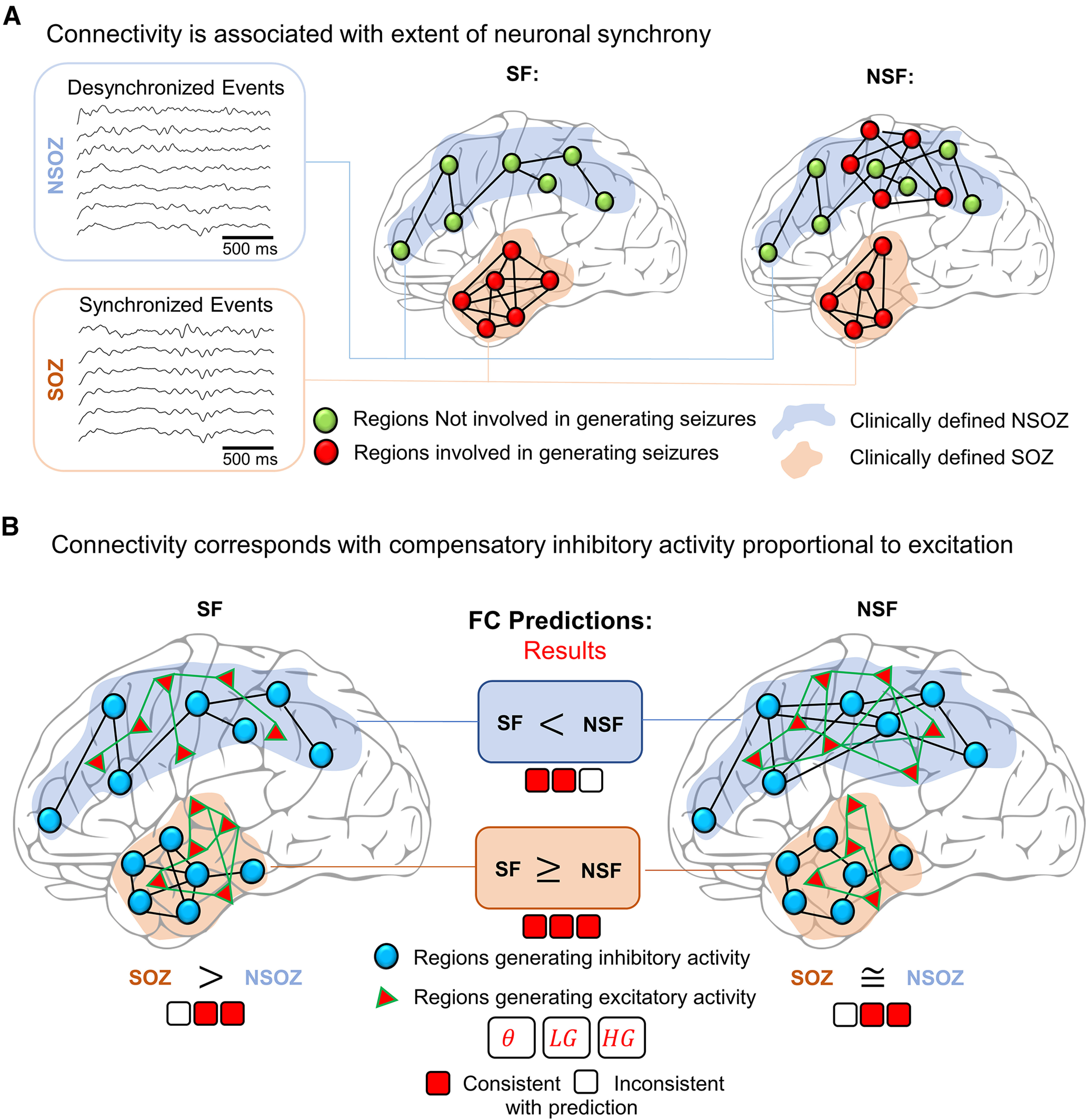
Relating connectivity to neuronal circuits mechanisms. ***A***, A schematic illustrating brain regions involved in generating seizures (red dots) or those not involved (green dots). Clinically-defined seizure onset zone (SOZ; shaded orange) and not seizure onset zone (NSOZ; shaded blue). In an ideal seizure outcome, i.e., seizure free, all regions involved in generating a seizure are in the SOZ. The synchrony between brain regions is illustrated as connections (black lines), and a greater number of lines indicates greater synchrony. In not seizure-free patients, the SOZ is incompletely identified and a portion of the NSOZ contains regions involved in generating seizures. ***B***, Prediction of the differences in the event connectivity when the strength of connectivity corresponds with increased synchronous inhibitory activity (blue dots and black lines) that is proportional to increased synchronous excitatory activity (red triangles and green lines). An assumption is greater synchrony associated with brain regions involved in generating seizures, which leads to the following predictions: (1) in seizure-free (SF) patients, stronger connectivity in SOZ than NSOZ; (2) in not seizure-free (NSF) patients, little or no difference in connectivity between SOZ and NSOZ; and (3) stronger connectivity in the NSOZ of NSF than SF patients. Results from our analysis are presented as three squares for each frequency band (theta = θ, low gamma = LG, high gamma = HG), which are colored red and white for actual results that are consistent or inconsistent, respectively, with the aforementioned predictions.

With this understanding of event connectivity, we interpret our results as follows. We assume that in seizure-free patients, brain regions corresponding with SOZ and NSOZ were completely identified, but in not seizure-free patients, the brain area responsible for generating seizures was incompletely identified and includes regions labeled SOZ and some in NSOZ (Fig. 8*A*). Prior work found increased excitability and synchrony in the SOZ (Staba et al., 2002; Schevon et al., 2007), and if this were because of deficits in inhibition, then it might be greater in not seizure-free than seizure-free patients to explain the recurrence of seizures. If this is correct and in the context of our current results, we should find stronger event connectivity in SOZ than NSOZ in seizure-free patients, which we do, and little difference between SOZ and NSOZ in not seizure-free patients, which also is consistent with our results. Furthermore, we should find weaker event connectivity, especially in NSOZ, of not seizure-free than seizure-free patients, but our results found stronger LGEC and ThEC in the NSOZ of not seizure-free patients. An alternative possibility is that rather than deficits in inhibition, there is a compensatory increase in the synchrony of inhibitory activity that is proportional to excitatory activity during interictal episodes (Fig. 8*B*). This explanation is more compatible with our results, particularly the stronger event connectivity in the NSOZ of not seizure-free patients and could correspond to increased synchrony of inhibitory and excitatory activity from an actual or potential SOZ (Lüders et al., 2006; Jehi, 2018).

### Frequency band-specific sensitivity for the SOZ

Analysis found more differences in γ-band than theta-band event connectivity. One reason could be that unlike theta activity in rats (Buzsáki, 2002), the mechanisms generating theta are unclear in humans. However, like rats, theta can be recorded from several subcortical and cortical areas, which we suggested could correspond with large or distributed neuronal sources. It is possible that some of our recording contacts recorded neuronal activity from a common theta source that overlapped with SOZ and NSOZ making it less sensitive to detect differences between SOZ and NSOZ than LGEC and HGEC. Also, we computed event connectivity from low-gamma and high-gamma bands like in previous rat studies (Kheiri et al., 2013; Bragin et al., 2014) and as is often done in studies on gamma (Buzsáki and Watson, 2012). Although we found similar results with LGEC and HGEC, they were not identical, and we plan future studies to investigate this further. Guiding this future work will be evidence suggesting that low gamma activity could involve inhibitory-inhibitory interactions and high gamma activity more dependent on inhibitory-excitatory interactions (Kay, 2003). The potential differences in the contribution of local (inhibitory) and projection cells (excitatory) between gamma and theta might be related to the differences we found in the correlation between strength of event connectivity and distance. The strength of LGEC declined more rapidly with longer distances than HGEC or ThEC, which could be explained by greater contributions of local inhibitory cells in the former and more involvement of projecting excitatory cells with the latter.

### Factors that could affect the strength of event connectivity

There are several factors to consider when interpreting the current results. First, it is important to distinguish between connectivity that derives from information theory and that based on amplitude or phase correlation/coherence methods. For example, in amplitude correlation, the power of the signal is an important factor that affects the strength of connectivity. As noted previously, event connectivity derives from the entropy of the peri-event histogram and is affected by the timing of the individual events. However, the algorithm detecting the peak of events requires an amplitude threshold, and it is possible it missed low amplitude events. Second, connectivity was computed from a 10- to 15-min interictal recording. Like previous studies, we selected a duration and time of recording to reduce potential effects of general anesthesia, spontaneous seizures, and anti-seizure medication tapering (Bartolomei et al., 2008a,b; Bettus et al., 2008; Medvedev et al., 2011; Cimbalnik et al., 2018; Lagarde et al., 2018; Klimes et al., 2019), and like these other studies, we found comparable results. Also, there is evidence that event connectivity is stable over a period of several days in freely behaving rats (Kheiri et al., 2013), and using the same methodology, we found 84.3 ± 13.0% (*n* = 5 patients) similarity in the strength of connectivity between signals from first 10 min and the last 10 min of the recording. Third, an increase in neuronal spiking firing during interictal spikes can generate γ activity (Alvarado-Rojas et al., 2013; Muldoon et al., 2015; Ren et al., 2015), which might overestimate the strength of connectivity on contacts with high rates of spikes. We included the rate of interictal spikes as a covariate in our linear mixed model and found spikes have a significant, but small, effect on connectivity. The latter result is consistent with other work that found little difference in connectivity values computed from EEG signals containing spikes and the same EEG signals after spikes were removed (Bettus et al., 2008). Fourth, we realize that Euclidean distance is an imprecise measure of anatomic connectivity, yet there was a significant effect of distance on events connectivity. Interelectrode distance was shorter in SOZ and NSOZ of seizure-free than not seizure-free patients, justifying the decision to include distance as a covariate in our linear mixed model. Same as for interictal spikes, connectivity values were adjusted for differences in distances and unlikely explain connectivity results with respect to seizure outcome. Lastly, measures of seizure severity, epilepsy burden, or other features of epilepsy did not correlate with the strength of connectivity after correcting for multiple comparisons, suggesting differences in connectivity with respect to SOZ and seizure outcome do not correspond with progressive aspects of epilepsy.

In conclusion, event connectivity is sensitive to differences in the synchrony of signals recorded in the SOZ and NSOZ and between surgical patients with and without seizure control. Differences in the strength of event connectivity between SOZ and NSOZ suggest a well-localized seizure network. By contrast, little or no difference in event connectivity could indicate a larger brain area generating seizures than localized to the SOZ and higher likelihood for seizure recurrence. In future work, we plan to perform unit recordings to investigate the neuronal basis of event connectivity and how changes in the strength of event connectivity correlate with neuronal excitability in brain areas where seizures begin and spread.

## Synthesis

Reviewing Editor: William Stacey, University of Michigan Decisions are customarily a result of the Reviewing Editor and the peer reviewers coming together and discussing their recommendations until a consensus is reached. When revisions are invited, a fact-based synthesis statement explaining their decision and outlining what is needed to prepare a revision will be listed below. The following reviewer(s) agreed to reveal their identity: Christos Papadelis, Ankit Khambhati.

This study corroborates the general hypothesis proposed by other network analytic studies conducted over the past decade -- that the seizure onset zone is functionally disconnected from surrounding brain areas in patients with focal epilepsy. Moreover, this study extends prior work by translating an event-based functional coupling measure derived from animal studies to human electrophysiology. An important outcome of this work is that the amount functional dysconnectivity of the seizure onset zone is associated with eventual clinical outcome after epilepsy surgery. However there are several areas for improvement.

1. The results should be presented better to explain how they relate to outcome. First, simply relating to SOZ is not sufficient, as there might be differences in how much of the SOZ was resected in different patients. That must be controlled, i.e. is the entire SOZ resected in every patient? Second, given the known large differences between TLE and extratemporal outcomes, the effect must clearly control for that difference as well. Third, can the results be presented in terms of a predictive tool to predict outcome? You have access to all the necessary information (i.e., a relatively large group of patients with both good and poor surgical outcomes, the delineation of the SOZ). This is the holy grail in the presurgical evaluation of epilepsy surgery.

2. The paper needs better organization. The abstract should clearly state the rationale, hypothesis and results. Statistical significance (or possibly estimation statistics) should be presented with final results. The rationale of the study is poorly stated. What is the main goal of the study, and which is the main hypothesis that is tested? The intro should frame the clinical situation better: the basic aspects of epilepsy surgery, define the epileptogenic zone vs SOZ, what is the main goal of epilepsy surgery and the challenges that the clinical team faces. Why new biomarkers of epilepsy are necessary? What do they have to offer? Why is important the use of more advanced signal processing tools?

3. What functional connectivity measures have been used and why, and why would this new tool be needed? This tool should be compared and framed with past FC tools described in the literature. GEC appears to be a novel metric that has not been thoroughly studied in human intracranial electrophysiology. Most functional connectivity studies in this space focus on linear, correlational measures based on spectral amplitude and phase. A quantification or discussion on theoretical grounds of the similarities and differences between GEC and more common metrics would help build further intuition for the reader.

4. The figures are presented out of order in the main text. To enhance readability I suggest re-numbering the figures or re-ordering the sections such that the figures are first presented/referenced in sequential order. It is indeed confusing for the reader to jump from Table T3 (line 69) to Figure 1A (line 98) then to Table T2 (line 136) to Figure 5-1 (line 160).

5. Electrodes from contacts placed within the white matter were excluded from further analysis. Were these electrographically silent? Were they resected after surgery? Please explain.

6. Please clarify the sampling rates used. There is mention of 200 Hz, resampling and downsampling to 1 kHz.

7. The selection of the data portions is unclear. Did the recordings contain frequent epileptiform discharges, or was frank epileptiform activity avoided?

8. How were the low-gamma and high-gamma frequency bands is odd (i.e., 30-55 Hz and 65-100 Hz) selected? Is this due to the notch filter at 60 Hz? It is unclear why only the theta and low-gamma and high-gamma bands were analyzed. If there is available literature that justifies this selection it should be provided?

9. Much attention has been placed on high-frequency oscillations (HFOs) as a potential biomarker of the true seizure onset zone. The present study compares GEC to spikes, but not to HFOs. Are we to interpret the GEC as a similar phenomenon as the HFO or are they distinct?

10. By extracting local peaks in the band-limited signal and quantifying the entropy of the per-event histogram between two channel pairs as a precursor step in calculating the GEC, it is possible you are effectively computing a cross-correlation between the amplitude envelope of two band-limited signals. Please comment on the possible relationship between GEC and amplitude correlation in the signal.

11. Instead of the correlation analysis relating the GEC connectivity matrix to the spike co-occurrence matrix, would a simpler analysis relating gamma/theta event occurrence to interictal spike occurrence on single channels (as opposed to channel pairs) yield a strong correlation?

12. The analysis examines event coupling matrices and spike co-occurrence during 10-15 minute windows for each patient.

a. Why was this window size chosen over shorter/longer windows?

b. To what extent are the results driven by the choice of a single arbitrary window? A bootstrap approach or random sampling approach might help demonstrate the robustness of the effect.

c. What brain state can we expect the patient to be in during the chosen window? Sleep/awake? Are time windows taken around the same time of day for each patient?

13. The line plot in Fig 4b is misleading as the ordering of the labels on the x-axis is arbitrary and the connected points in the plot are intended to convey a sequential order to the effect. We suggest swapping the line plot for a bar plot to depict effect size.

14. Could the small effect size relating spikes to HGEC be due to the chosen window? Especially considering that spike rate can be variable over time, is it possible that the chosen windows just happen to be periods with low number of spikes?

15. The discussion section should be organized better, perhaps with some clear headings to describe the subject matter, or more correlated with the logical flow of the results section. Currently it is somewhat haphazard.

16. What is SOZc? Isn’t that just “non-SOZ” or “NSOZ”? This is difficult labeling and should be replaced with more standard notation.

17. The acronym “SF” is unknown and confusing. Just write out “seizure free” and “non-seizure free"
