# Supplementary figures and images for: Interictal Gamma Event Connectivity Differentiates the Seizure Network and Outcome in Patients after Temporal Lobe Epilepsy Surgery
Source: eNeuro. 2022 Dec 15;9(6):ENEURO.0141-22.2022. doi: 10.1523/ENEURO.0141-22.2022 (PMC9770020; doi:10.1523/ENEURO.0141-22.2022)

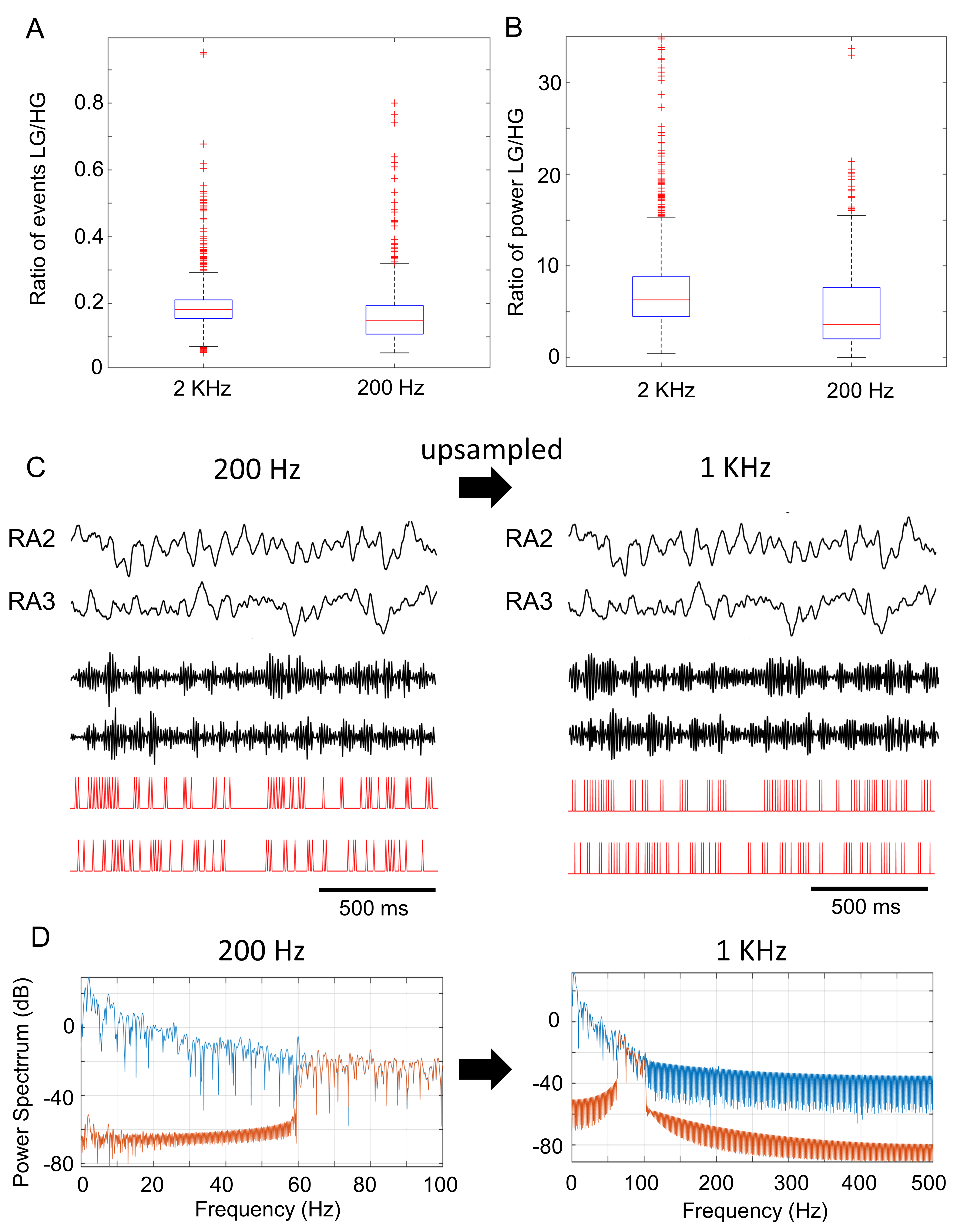

Supplement: Extended Data Figure 1-1 — Effect of low sampling and up-sampling on HGEC. A, Ratio of low γ events to numbers of high γ events is box-plotted for patients with 2-kHz sampling (N = 15) and those with 200-Hz sampling rate (N = 28). B, Same as A but for ratio of powers instead of ratio of number of events. We calculated those measures on five channels randomly selected from each patient on a randomly selected 30-s window and repeated the procedure 10 times. In total, we had 750=15×5×10 datapoints for the patients sampled at 200 Hz, and 1400=28×5×10 datapoints for the patients sampled at 2 kHz. C, Two signals extracted from the right amygdala for first patient in Table 3 sampled at 200 Hz (left) are illustrated with their corresponding high γ band-filtered signals (65–95 Hz) and train of high γ events are presented underneath. The up-sampled signals (1 kHz) and train event is present to the right. D, Power spectrum for the raw signal (RA2 in C, blue) and for the filtered signal (orange) are presented. The power spectrum of the up-sampled signal is presented to the right. Download Figure 1-1, TIF file. [file enu-eN-NWR-0141-22-s02.tif]

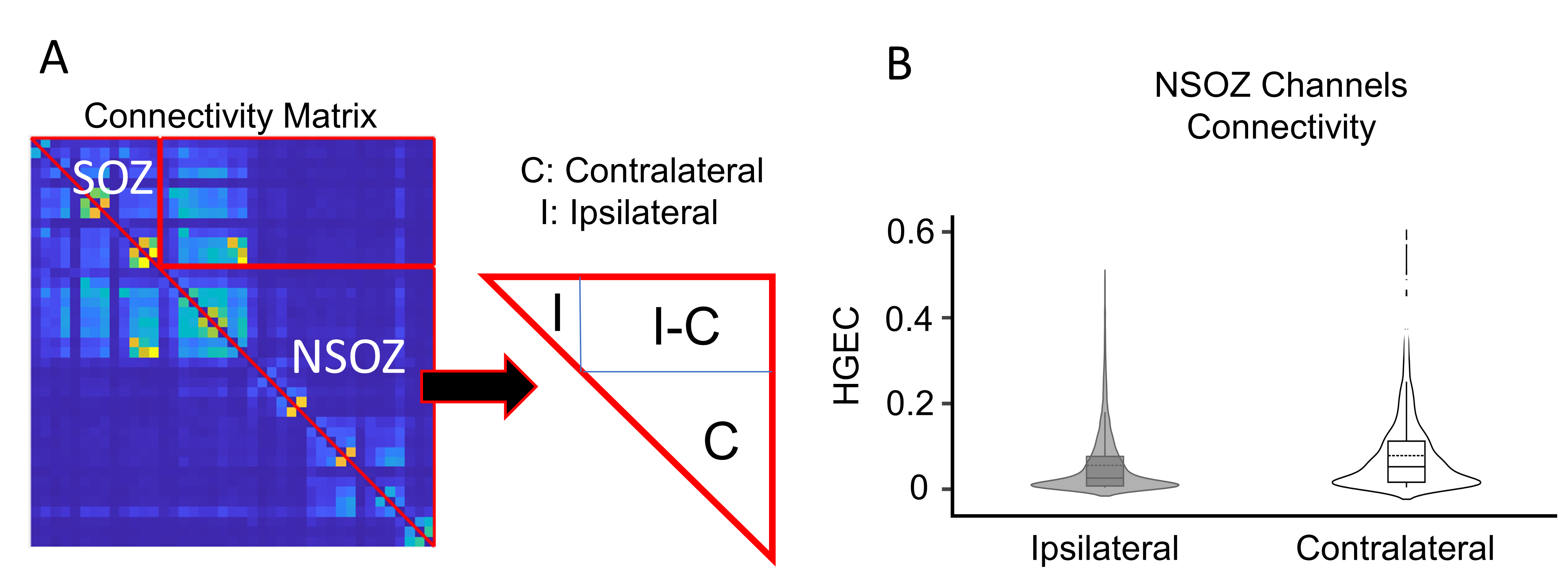

Supplement: Extended Data Figure 1-2 — HGEC in NSOZ ipsilateral versus contralateral. Comparison between HGEC connectivity values in channels located outside the SOZ but in same hemisphere (ipsilateral) and those in the opposite hemisphere (contralateral). A, Connectivity matrix where the NSOZ is organized by connectivity within the ipsilateral hemisphere (I), contralateral hemisphere (C), and between ipsilateral and contralateral hemispheres (I-C). B, Boxplots illustrate the connectivity values of ipsilateral NSOZ channels and contralateral NSOZ channels. No significant difference was obtained (effect size η2<0.01). Download Figure 1-2, TIF file. [file enu-eN-NWR-0141-22-s03.tif]

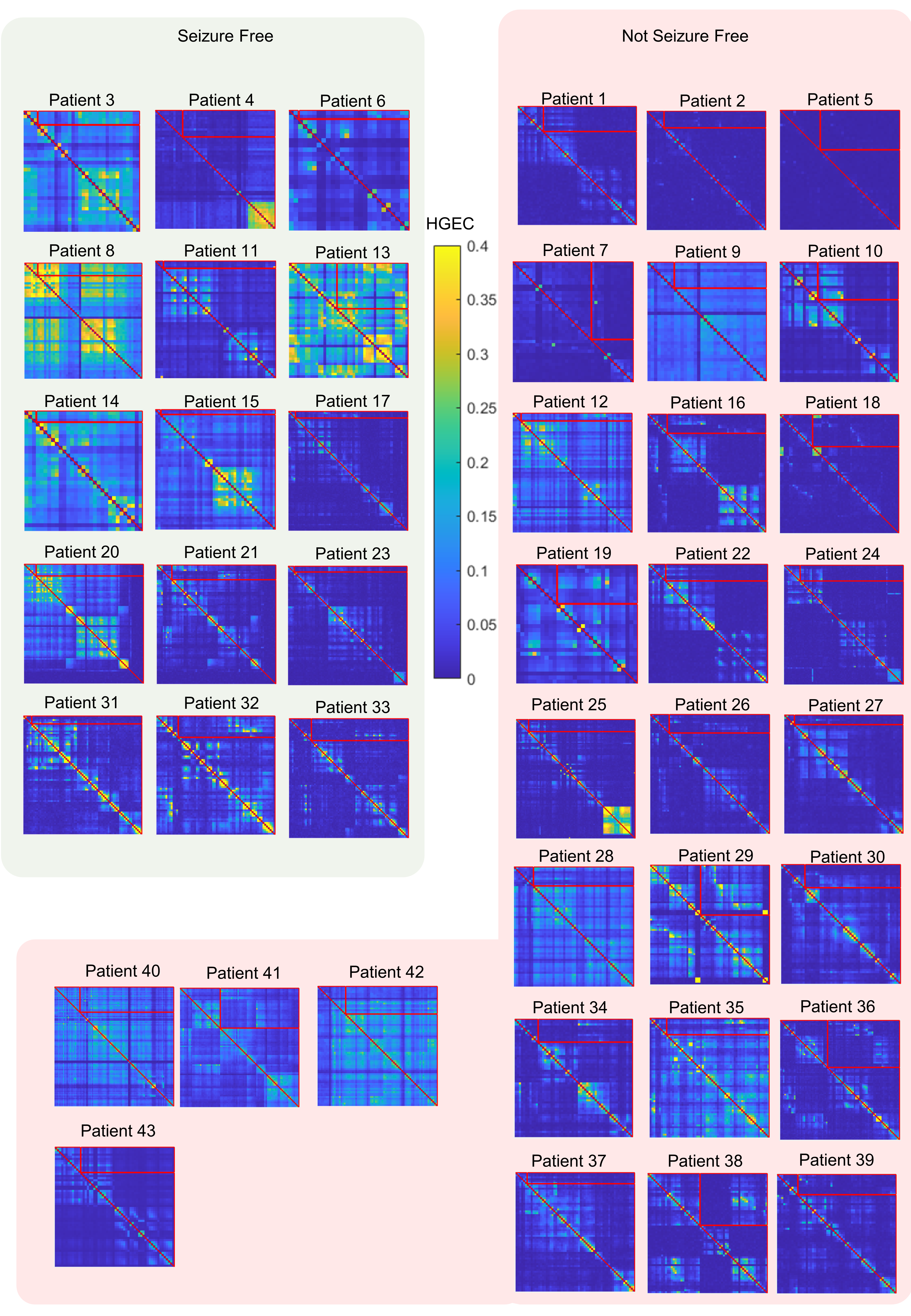

Supplement: Extended Data Figure 2-1 — High γ events connectivity (HGEC) matrices for all seizure-free patients (green) and not seizure-free patients (red) are presented. The matrices are organized by connectivity within the seizure onset zone (SOZ; upper triangle), within the seizure onset zone complement (NSOZ; lower triangle) and between the SOZ and NSOZ (rectangle) networks. Download Figure 2-1, TIF file. [file enu-eN-NWR-0141-22-s04.tif]

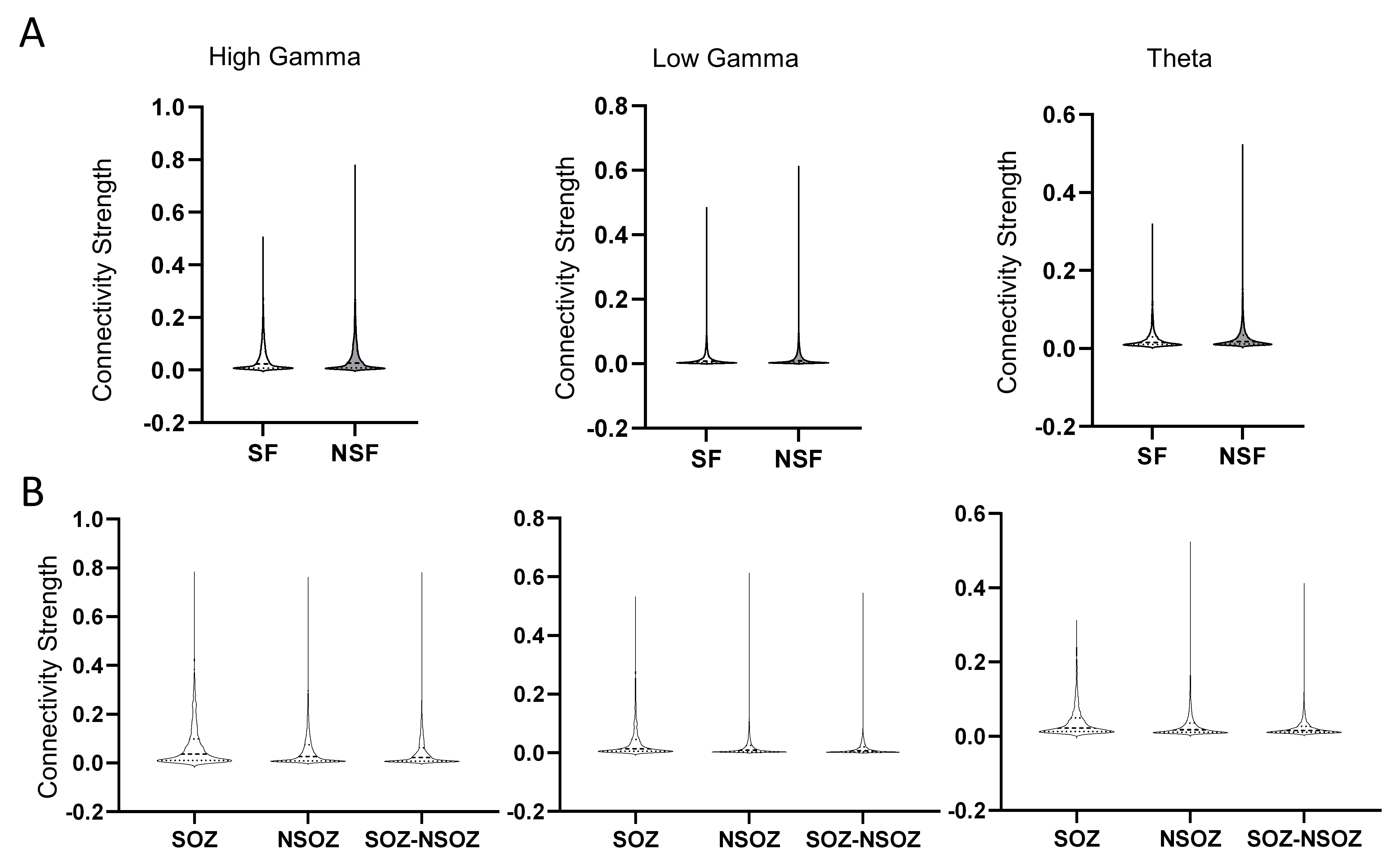

Supplement: Extended Data Figure 3-1 — Connectivity strength in relation to seizure outcome or SOZ. A, Violin plots that show HGEC, LGEC, and ThEC in relation to seizure outcome where SF patients are shaded in white and NSF are shaded in black. B, Violin plots that show HGEC, LGEC, and ThEC in relation to SOZ. Download Figure 3-1, TIF file. [file enu-eN-NWR-0141-22-s05.tif]

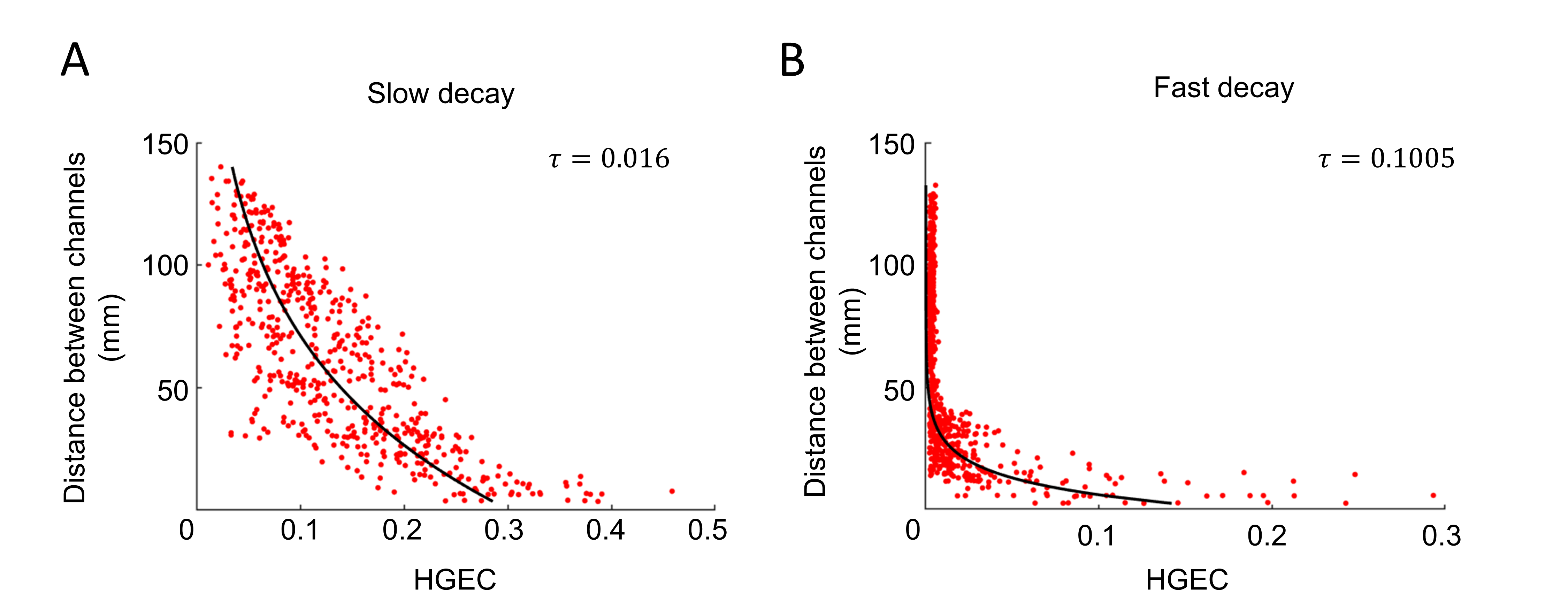

Supplement: Extended Data Figure 6-1 — The exponential relationship between HEC and Euclidian distance is plotted slow decaying HGEC (A, patient 3) and fast decaying HGEC (B, patient 24). Download Figure 6-1, TIF file. [file enu-eN-NWR-0141-22-s06.tif]

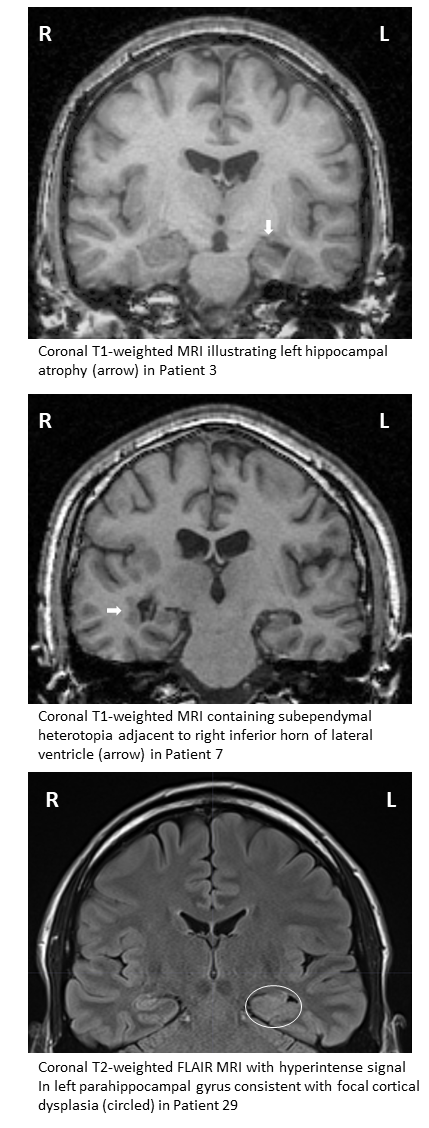

Supplement: Extended Data Figure 7-1 — Representative MRI from three patients in this study illustrating the different types of MRI pathology found in these cases that required invasive EEG. Download Figure 7-1, TIF file. [file enu-eN-NWR-0141-22-s07.tif]

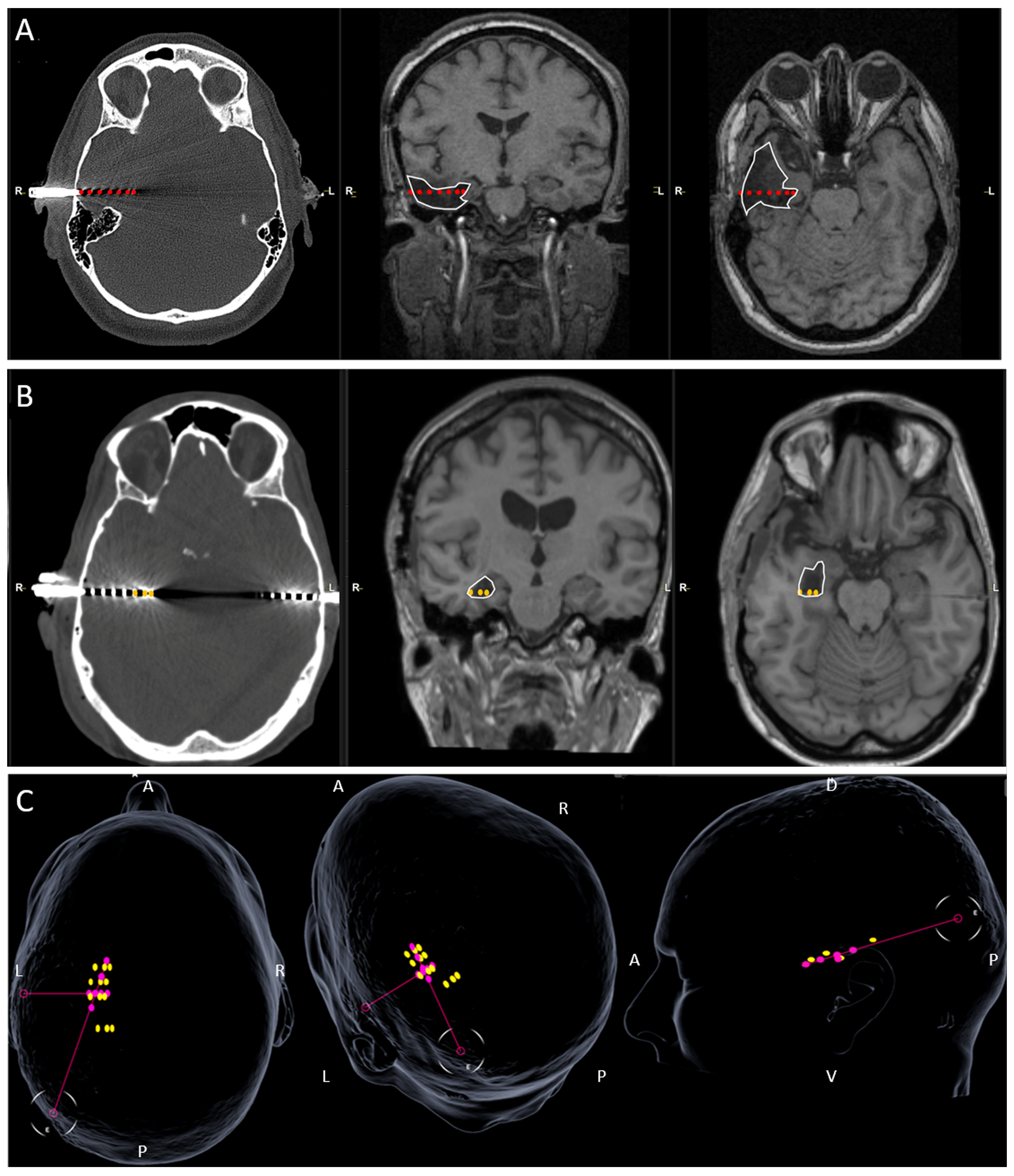

Supplement: Extended Data Figure T3-1 — Resection or RNS therapy in the SOZ. A, Resection of tissue corresponding to SOZ in patient 24. Postimplant CT (left, axial) registered with postsurgical MRI in coronal (middle) and axial planes (right). Red dots denote contacts of depth electrode with distal contacts positioned in right entorhinal cortex. Area outlined in white indicates the margins of resection in the plane of view. B, Same as panel A, but patient 39 and yellow dots denote contacts of depth electrode positioned to sample right middle hippocampus. C, RNS therapy of the left mesial temporal lobe SOZ, including entorhinal cortex, in patient 35. Full-head model illustrates trajectories of two RNS probes (magenta lines) with one entry (E) from occipital cortex with contacts (magenta dots) positioned in left amygdala, hippocampus, and parahippocampal gyrus, and the other E from lateral aspect of temporal cortex with contacts in and adjacent to entorhinal cortex. Yellow dots denote depth electrode contacts of the left SOZ involving amygdala, entorhinal cortex, middle hippocampus, and parahippocampal gyrus. Sagittal view (top), clockwise-rotated posterolateral view (middle), and axial view (bottom). A = anterior, P = posterior, D = dorsal, V = ventral, L = left, and R = right. Download Figure T3-1, TIF file. [file enu-eN-NWR-0141-22-s08.tif]
